# Design, Synthesis and Characterization of Cyclic NU172 Analogues: A Biophysical and Biological Insight

**DOI:** 10.3390/ijms21113860

**Published:** 2020-05-29

**Authors:** Claudia Riccardi, Albert Meyer, Jean-Jacques Vasseur, Domenico Cavasso, Irene Russo Krauss, Luigi Paduano, François Morvan, Daniela Montesarchio

**Affiliations:** 1 Department of Chemical Sciences, University of Naples Federico II, Via Cintia 21, I-80126 Naples, Italy; claudia.riccardi@unina.it (C.R.); domenicocavasso@gmail.com (D.C.); irene.russokrauss@unina.it (I.R.K.); luigi.paduano@unina.it (L.P.); 2 Institut des Biomolécules Max Mousseron, Univ. Montpellier, CNRS, ENSCM, 34095 Montpellier, France; albert.meyer@umontpellier.fr (A.M.); jean-jacques.vasseur@umontpellier.fr (J.-J.V.); 3 CSGI—Consorzio Interuniversitario per lo Sviluppo dei Sistemi a Grande Interfase, Via della Lastruccia 3, I-50019 Sesto Fiorentino, Italy

**Keywords:** DNA aptamers, G-quadruplex, duplex/quadruplex, NU172, cyclization, biophysical characterization, structure-activity relationship, thrombin, anticoagulant activity

## Abstract

NU172—a 26-mer oligonucleotide able to bind exosite I of human thrombin and inhibit its activity—was the first aptamer to reach Phase II clinical studies as an anticoagulant in heart disease treatments. With the aim of favoring its functional duplex-quadruplex conformation and thus improving its enzymatic stability, as well as its thrombin inhibitory activity, herein a focused set of cyclic NU172 analogues—obtained by connecting its 5′- and 3′-extremities with flexible linkers—was synthesized. Two different chemical approaches were exploited in the cyclization procedure, one based on the oxime ligation method and the other on Cu(I)-assisted azide-alkyne cycloaddition (CuAAC), affording NU172 analogues including circularizing linkers with different length and chemical nature. The resulting cyclic NU172 derivatives were characterized using several biophysical techniques (ultraviolet (UV) and circular dichroism (CD) spectroscopies, gel electrophoresis) and then investigated for their serum resistance and anticoagulant activity in vitro. All the cyclic NU172 analogues showed higher thermal stability and nuclease resistance compared to unmodified NU172. These favorable properties were, however, associated with reduced—even though still significant—anticoagulant activity, suggesting that the conformational constraints introduced upon cyclization were somehow detrimental for protein recognition. These results provide useful information for the design of improved analogues of NU172 and related duplex-quadruplex structures.

## 1. Introduction

Thrombin is a multifunctional “trypsin-like” serine protease that plays a fundamental role in the last step of blood clotting, able to catalyze the conversion of soluble fibrinogen into insoluble fibrin strands [1,2,3,4,5,6], also intervening in other physiological and pathological processes [7,8,9]. Despite its pivotal function, thrombin activity needs to be repressed in some cases, e.g., coronary surgery, cancer therapy, treatment of cardiovascular diseases, to name just a few. For instance, the inhibition of its functionality is one of the most effective antithrombotic strategies and therefore the development of efficient anticoagulant agents is of paramount importance [10,11,12,13,14].

**Figure 1:**
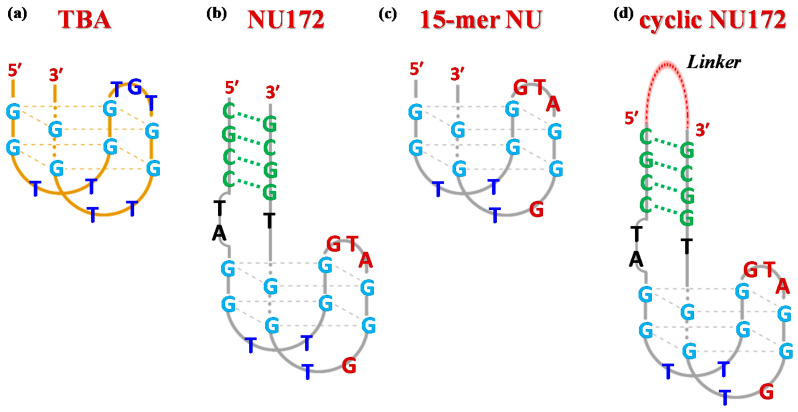
Schematic representation of the antiparallel G-quadruplex structure of TBA (**a**), the duplex/quadruplex structure of NU172 (**b**), its minimal G4 motif indicated as NU (**c**) and a general scheme for cyclic NU172 analogues (**d**) with a linker connecting the 3′- and 5′-ends of the oligonucleotide sequence. The guanines involved in the G-quadruplex core formation are highlighted in light blue, while the duplex module is depicted in green. In both NU and NU172 structures, the nucleobases of the G4 core differing from TBA are indicated in dark red.

Currently, the 15-mer G-rich oligonucleotide, named Thrombin Binding Aptamer (TBA, also known as HD1)—with the sequence 5′-GGTTGGTGTGGTTGG-3′, Figure 1a—is one of the most extensively studied systems among known thrombin-targeting agents [13,15,16,17]. Upon folding into an antiparallel, intramolecular G-quadruplex (G4) structure with a chair-like conformation [18,19], TBA can efficiently act as a thrombin activity modulator, being able to selectively recognize the fibrinogen-binding exosite I of the protein [20], thus inhibiting fibrin clot formation [21,22,23].

Unfortunately, due to the high doses needed to achieve satisfactory therapeutic effects, TBA did not progress over Phase I studies in clinical trials [21,24]. Nevertheless, its high potential for therapeutic [25,26,27] and diagnostic [28,29] applications stimulated the development of novel TBA analogues designed to improve its overall properties and overcome the observed drawbacks. Following this approach, a large number of chemically modified TBA variants have been proposed in the literature [15]. These efforts have led to several optimized analogues, showing remarkably improved physico-chemical and biological properties, although none of them have reached advanced clinical trial studies thus far [13,30,31].

As a general strategy to obtain TBA analogues better performing in vitro and in vivo, we recently explored a cyclization approach [32,33]. By chemically linking the extremities of TBA, we aimed at stabilizing its functional G4 folding, in parallel capping its 5′ and 3′-ends so to ensure enhanced resistance to nuclease digestion. We thus synthesized a mini library of cyclic TBA derivatives, obtained by covalently connecting the aptamer ends with different flexible linkers [32,33]. A fine-tuning of the length and chemical nature of the linker allowed identifying a cyclic analogue, named cycTBA II, which—compared to the native linear aptamer—showed higher anticoagulant activity and enzymatic resistance in serum, as well as a remarkably stabilized G4 structure [33].

These encouraging results suggested that the cyclization strategy could be successfully extended also to other G4-forming aptamers in order to improve their biophysical and/or biological properties.

In this frame, we considered particularly valuable to include in our studies also bimodular aptamers, encompassing both duplex and G4 domains. These second-generation anticoagulant aptamers are widely investigated for biomedical applications due to their more specific and effective activity than molecules featuring only a single, either a duplex or a G4, structural motif [34,35].

Indeed, in the family of known anticoagulant aptamers, TBA represents the minimal pharmacophore necessary for high-affinity and highly specific recognition of thrombin. The addition of further structural elements to the G4 core of TBA proved to be successful in improving its thrombin recognition abilities, enhancing its affinity for the protein [36,37] and/or inhibitory activity [38].

Among the anti-thrombin bimodular oligonucleotides, NU172—a 26-mer with the sequence 5′-CGCCTAGGTTGGGTAGGGTGGTGGCG-3′, Figure 1b [39]—entered Phase II clinical trials as an anticoagulant in heart disease treatments [40]. This mixed duplex-quadruplex aptamer—discovered using Systematic Evolution of Ligands by EXponential enrichment (SELEX) strategy [41]—showed short-acting anticoagulation properties, also being well tolerated without serious side effects [27]. Notably, NU172 exhibited an IC_50_ value of 5–10 µg/mL in plasma in an ecarin clotting time (ECT) assay, as determined by thromboelastography [42].

From a structural point of view, NU172 is characterized by a duplex module combined with a minimal G-quadruplex motif, whose primary sequence differs from that of TBA not only for the additional duplex domain—composed of four pairs of complementary nucleotides—and the residues connecting the duplex and the quadruplex motifs (connecting residues), but also for the loops of the G4 core. In detail, the TBA-like G4 motif—i.e., 5′-GGTTGGGTAGGGTGG-3′, here indicated as NU—differs from that of TBA by three nucleotides in the central loop (GTA in place of TGT) and by one nucleotide in the 3′ lateral loop (GT in place of TT, Figure 1c). The different structure of NU172 compared to that of TBA is definitively responsible for superior anti-thrombotic activities [43,44], as well as an improved affinity towards the target protein [45,46], for which it also represents a promising bio-recognition tool in the context of high performance thrombin aptasensors [47].

Preliminary biophysical investigations on NU172 demonstrated a strong mutual influence of each domain on the stability of the other [48,49,50,51]. More recently, Troisi et al. solved the crystallographic structure of the thrombin-NU172 complex, providing a deep insight into the aptamer folding and its binding to thrombin exosite I, which takes advantage of all three loops of the G4 module [52], due to a peculiar conformation involving the connecting residues as well. Even if the duplex domain does not directly interact with the protein, nonetheless it plays a pivotal role in the modulation of the NU172 properties, strongly influencing the thrombin-aptamer recognition and subsequently the bioactivity [52], in line with previous observations on other bimodular aptamers [37,53]. In particular, the comparison with NU, corresponding to the minimal G4 of NU172, indicated that the presence of the duplex tract was crucial in determining the G4 loops conformation, on which NU172 anticoagulant activity depends [52].

Considering the main role of the duplex domain in the structure/activity relationships of NU172 [52], we reasoned that by connecting its extremities with ad hoc designed linkers—as schematically depicted in Figure 1d—a favorable impact on the biological properties of this aptamer could be achieved, as already found for TBA [32,33]. The basic idea was to obtain improved NU172 analogues through a good compromise between increasing the aptamer compactness, thus “freezing” its functional conformation, and maintaining some structural flexibility, somehow crucial for thrombin recognition. In addition, by covalently capping the 3′- and 5′-ends of the oligonucleotide chain with a suitable linker, two additional advantages were expected, i.e., a marked stabilization to nuclease degradation—which is the major limitation to in vivo usage of anticoagulant aptamers—and a net reduction of oligomerization events, reported in the literature for G-rich duplex/quadruplex constructs [50].

Herein, we report the design, synthesis and characterization of cyclic derivatives of NU172, in which the oligonucleotide chain was circularized exploiting two different chemical strategies. By using several biophysical techniques, the influence of the different inserted linkers on the conformational behavior, thermal stability and nuclease resistance of the cyclic NU172 variants was analyzed in comparison with unmodified NU172. In parallel, with the aim of better understanding the effect of the duplex domain on the NU172 behavior, also NU, corresponding to the minimal G4 motif of the bimodular aptamer, was studied as a reference oligonucleotide. Finally, light scattering (LS) analyses were carried out to determine the anticoagulant activity of the cyclic analogues compared to unmodified NU172 and NU oligonucleotides.

## 2. Results and Discussion

### 2.1. Design of the Cyclic NU172 Derivatives

Stimulated by our previous investigations on cyclic TBAs [32,33], we designed a small library of cyclic NU172 analogues (Table 1), containing different flexible linkers connecting the 3′ and 5′ oligonucleotide extremities. The selected linkers vary for their length and chemical properties, thus allowing to evaluate their impact on the overall aptamer properties. All the designed linkers spanned similar lengths, in the range of 23–27 atoms, considered—on the basis of available crystallographic data [52]—suitable to keep the extremities of NU172 at a proper distance, not impairing the optimal folding necessary for thrombin recognition.

In detail, two cyclic derivatives, here indicated as **cycNU172-EG2** and **cycNU172-EG3**, were circularized using a Cu(I)-assisted azide-alkyne cycloaddition (CuAAC) protocol [54]. They differ for the length of the oligoethylene glycol chain, thus providing 24- and 27-atoms long linkers, respectively for **cycNU172-EG2** and **cycNU172-EG3** (Table 1).

The other two analogues, here named **cycNU172-Ph** and **cycNU172-Pro**, were obtained using a chemical cyclization strategy based on a bis-oxime ligation method [55,56], producing the target derivatives with linkers of similar length (24- vs. 23-atoms long linkers, respectively for **cycNU172-Ph** and **cycNU172-Pro**, Table 1) but distinct chemical natures due to the different bis-aldehyde, i.e., terephthalaldehyde and glutaraldehyde, exploited in the cyclization procedure.

As a consequence, **cycNU172-Ph** included a phenyl ring in its linker, while in **cycNU172-Pro** a purely aliphatic and more flexible connection was present (Table 1). In turn, **cycNU172-EG2** and **cycNU172-Ph**, sharing a linker of the same length (i.e., 24 atoms, Table 1) but of different chemical structure, allowed for a better understanding of the influence of the linker on the aptamer behavior.

With the design of these cyclic derivatives of NU172, we intended to obtain suitable analogues with high potency in inhibiting fibrin clot formation, and concomitantly increased G4 conformational stability and/or nuclease resistance.

### 2.2. Synthetic Procedures for the Preparation of Cyclic NU172 Variants and Their Characterization

Both **cycNU172-EG2** and **cycNU172-EG3** were synthesized using CuAAC cyclization, following a previously described procedure [32,33,54]. The oligonucleotide sequence was elongated on the azido-functionalized solid support **1** [54] and propargyl-diethyleneglycol **2** [57] or propargyl-triethyleneglycol **3** [58] phosphoramidites were used for the last coupling step, respectively affording compounds **4** and **5** (Scheme S1). After a standard ammonia deprotection, the crude linear oligonucleotides **6** and **7** were cyclized using CuSO_4_ and sodium ascorbate in the presence of KCl, necessary to promote the G4 formation. The reaction mixtures—monitored by high performance liquid chromatography (HPLC) analysis over time (Figures S1–S3)—showed an almost complete formation of the target products after 4 h, which exhibited lower retention times than the linear oligonucleotides, nicely confirming their effective circularization [32,33,54]. These mixtures were desalted by size exclusion chromatography (SEC) and then purified by reverse phase C18 HPLC, giving **cycNU172-EG2** and **cycNU172-EG3** (Figures S2 and S3).

The other two cyclic NU172 analogues of the series, **cycNU172-Ph** and **cycNU172-Pro**, were prepared by bis-oxime ligation [32,33,56]. The elongation of the NU172 sequence was performed starting from the ethyleneglycol oxime-functionalized solid support **8** [59] and, after the oligonucleotide sequence assembly, MMTr-aminooxytriethyleneglycol phosphoramidite **9** [33] was used for the last coupling step (Scheme S2). A first treatment with 7 M methanolic ammonia, followed by a second treatment with methoxyamine, allowed for the deprotection and release from the solid support of the oligonucleotide anchored in **10**, affording the linear oligonucleotide **11**, equipped with an aminooxy function at each end. The cyclization step was then performed treating **11** with terephthalaldehyde or glutaraldehyde in ammonium acetate buffer in the presence of KCl. The reactions were monitored by HPLC analyses (Figures S4–S6) and the target cyclic oligonucleotides, i.e., **cycNU172-Ph** and **cycNU172-Pro**, were then purified by reverse phase HPLC. The cyclization reactions of these oligonucleotides were slower than previously observed for the cyclic TBA series [32,33] and the crudes were also less pure, with consequent lower yields for the final cycNU172 derivatives.

All the cyclic NU172 analogues and their oligonucleotide precursors were analyzed by HPLC and characterized by matrix-assisted laser desorption/ionization-time of flight mass spectrometry, i.e., MALDI-TOF MS (Figures S1–S6).

To further check their purity, a 20% denaturing polyacrylamide gel electrophoresis (PAGE) analysis was carried out, in comparison with native NU172 and NU, here used as reference oligonucleotides (Figure S7). In denaturing conditions, all the investigated sequences migrated as a single band on the gel, showing very similar electrophoretic mobility for NU172 and its cyclic derivatives—as expected, for their comparable mass/charge ratios—and faster mobility for the smaller NU.

### 2.3. Conformational and Spectroscopic Properties of Cyclic NU172 Variants

#### 2.3.1. UV Spectroscopy Analysis: Thermal Difference Spectra and UV Thermal Denaturation/Renaturation Measurements

The spectroscopic properties and conformational behavior of the cyclic NU172 derivatives were investigated by UV and CD analyses in comparison with unmodified NU172 and NU. In order to evaluate the effects of different saline conditions on the structuring ability of these novel cyclic aptamers, the biophysical investigation was carried out in two different phosphate buffer solutions, containing a high content of K^+^ (10 mM KH_2_PO_4_/K_2_HPO_4_, 70 mM KCl, 0.2 mM EDTA, pH 7.3, here indicated as K^+^-rich buffer) or of Na^+^ (i.e., PBS: 137 mM NaCl, 2.7 mM KCl, 10 mM NaH_2_PO_4_/Na_2_HPO_4_, 1.8 mM KH_2_PO_4_/K_2_HPO_4_, pH 7.3, here indicated as Na^+^-rich buffer) ions, respectively mimicking the intra- and extracellular environment. It is indeed well known that buffer composition, and particularly ion concentration, can affect the biophysical properties and functional activity of selected aptamers, playing a key role in determining the secondary structure and stability of their G4 folding [48,60,61,62].

**Figure 2:**
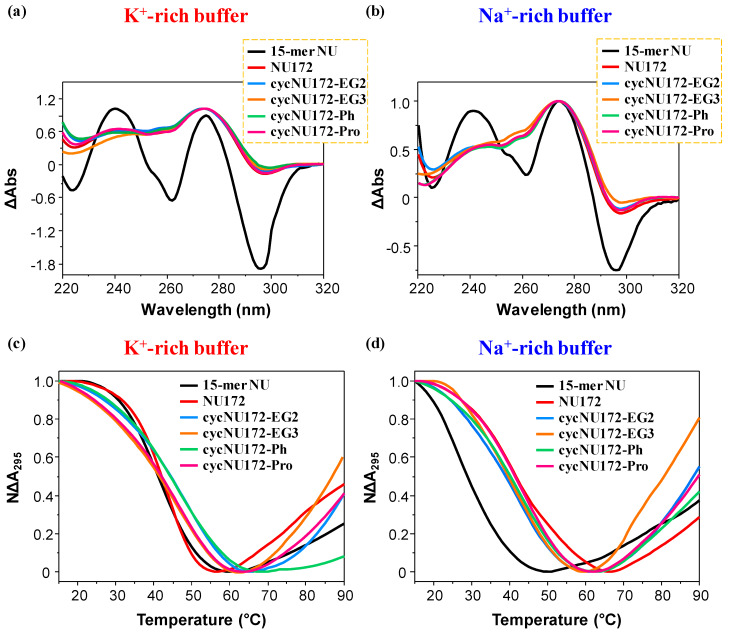
Representative normalized TDS (**a**,**b**) and UV-melting profiles (**c**,**d**) of the cyclic NU172 derivatives (light blue, orange, green, and magenta lines, respectively for **cycNU172-EG2**, **cycNU172-EG3**, **cycNU172-Ph** and **cycNU172-Pro**). All the oligonucleotide samples were analyzed at 2 µM concentration in both the selected K^+^- (**a**,**c**) and Na^+^-rich (**b**,**d**) buffer solutions, in comparison with unmodified NU172 and NU (dark red and black lines, respectively). TDS profiles result from the subtraction of the UV spectrum registered at 15 °C from the 90 °C one. UV-melting profiles—recorded at 295 nm using a scan rate of 1 °C/min—are reported as normalized absorbance as a function of the temperature.

For all the oligonucleotides, UV thermal difference spectra (TDS, Figure 2a,b) were obtained recording the UV spectra at low and high temperatures (i.e., 15 and 90 °C) at 2 μM concentration. The spectral difference between the unfolded and folded oligonucleotide represent indeed a typical “fingerprint”, useful to discriminate different classes of nucleic acid structures [63,64,65,66].

The normalized TDS profiles of NU (Figure 2a,b, black line) were similar in both buffer solutions, with two positive (around 240 and 270 nm) and two negative (at ca. 260 and 295 nm) bands, diagnostic of a G4 structure [63]. For this compound, TDS factors could be calculated, allowing for the estimation of the predominant G4 folding in solution. Three TDS factors were determined, with values lower than 2, 1.5 and 2 respectively for the ΔA_240_/ΔA_295_, ΔA_255_/ΔA_295_ and ΔA_275_/ΔA_295_ absorbance ratios (Table S1), all consistent with an antiparallel G4 topology, in accordance with literature data [65].

In turn, the TDS profiles of NU172 showed the maximum and minimum, respectively, around 270 and 295 nm, but not the minimum at 260 nm, while the maximum around 240 nm was less pronounced (Figure 2a,b, dark red line). The peculiar TDS profile found for NU172 in both the explored saline conditions was consistent with that of a duplex/quadruplex structure, containing an antiparallel G4 conformation for the G4 module, as also previously found for other anticoagulant bimodular aptamers [67].

The TDS profiles of all the cyclic NU172 analogues were very similar to that of unmodified NU172, indicating that the introduction of the circularizing linker had no dramatic influence on the overall duplex/quadruplex folding (Figure 2a,b, light blue, orange, green, and magenta lines, respectively for **cycNU172-EG2**, **cycNU172-EG3**, **cycNU172-Ph** and **cycNU172-Pro**), also denoting similar features in both the examined buffers.

Then, UV-monitored thermal denaturation/renaturation experiments were performed to study the thermal stability of both the G-quadruplex and duplex domains of the cyclic NU172 analogues in comparison with NU172 and its “nude” G4 motif NU. It was previously shown, in fact, that the UV-melting curves monitored at 295 and 260 nm give information respectively on the unfolding of the G-quadruplex and duplex domains of the bimodular aptamers [49,50,51,68].

All these experiments were performed at 2 µM oligonucleotide conc. in the 15–90 °C temperature range. In Figure 2 and Figure S8, the overlapped UV-melting profiles following the absorbance changes on increasing the temperature, respectively at 295 and 260 nm, are reported, while in Figures S9–S14 the overlapped UV-monitored heating and cooling curves for each compound are shown at both wavelengths. All the T_m_ values obtained from these UV analyses, determined as the temperatures at which the normalized absorbance of each compound is equal to 0.5, are summarized in Table S2.

For all the oligonucleotides, a sigmoidal decrease of the absorbance at 295 nm was found upon increasing the temperature (Figure 2, panel c and d, respectively for the K^+^- and Na^+^-rich buffers), in agreement with the unfolding of the G4 domain [66,69,70], while the heating processes monitored at 260 nm (Figure S8, panel a and b, respectively for the K^+^- and Na^+^-rich buffers) produced hyperchromic effects upon melting, as generally observed for duplex unfolding [49,50,51,68].

In particular, in the UV-thermal denaturation analysis at 295 nm, in the presence of K^+^ ions, NU and NU172 produced similar UV-melting curves (Figure 2c, Figures S9a and S10a) with nice sigmoidal profiles and similar T_m_ values within the experimental error (Table S2). On the contrary, in the Na^+^-rich phosphate buffer, NU172 (Figure 2d and Figure S10b) denoted an apparent T_m_ value of 41 °C (Table S2), in good accordance with previous results in similar saline conditions [49,50], showing an enhanced thermal stability compared to NU, for which a T_m_ of 29 °C was obtained (Figure 2d, Figure S9b and Table S2). Thus, if in the presence of K^+^ ions, NU172 and NU—sharing the same G4-forming sequence, unfolded approximately at the same temperature, in the Na^+^-rich medium—the G4 structure of NU172 was markedly more stable compared to NU. These results suggested that, in these saline conditions, the duplex motif in NU172 was able to stabilize the aptamer G4 folding, partially counterbalancing the destabilization of the G4 domain occurring in the presence of Na^+^ ions, as already observed for other G4 and mixed duplex/G-quadruplex architectures [49].

In the presence of K^+^ ions, all the cyclic NU172 variants showed higher (for **cycNU172-EG2** and **cycNU172-Ph**, Figure 2c, Figures S11a, S13a and Table S2) or comparable (for **cycNU172-EG3** and **cycNU172-Pro**, Figure 2c, Figures S12a, S14a and Table S2) T_m_ values than linear NU172.

Conversely, in the Na^+^-rich solution (Figure 2d, Figures S11 and S14b), only **cycNU172-Pro** denoted G4 thermal stability comparable to NU172, while a slight destabilization was found for the other cyclic derivatives (Table S2). Hence, in both saline conditions, UV analysis did not reveal substantial differences in the thermal stability of the G4 module of the here investigated cyclic NU172 analogues compared to their natural counterpart (Figure 2c,d and Table S2).

Considering the UV-heating and cooling experiments monitored at 295 nm, in almost all cases, a nice sigmoidal behavior was found (Figures S9–S14, panels a and b), with no significant hysteresis between melting and annealing profiles, suggesting that, under the explored experimental set up (scan rate = 1 °C/min), the related unfolding/folding processes were essentially reversible.

When monitoring the UV-thermal denaturation experiments at 260 nm (Figures S8, S10–S14), neither NU172 nor its cyclic analogues showed nice sigmoidal profiles, and the heating and cooling profiles were superimposable in no case, indicating that these processes were not reversible under the explored conditions.

NU172 showed T_m_ values of 60 and 58 °C, respectively in the K^+^ and Na^+^-rich buffer solutions (Figures S8, S10c,d and Table S2), in accordance with previous reports [50]. In both the explored saline conditions, all the cyclic derivatives indicated remarkably enhanced thermal stability compared to unmodified NU172, although with some differences (Figures S8, S10–S14 and Table S2).

Following the absorbance changes at 260 nm, the overall UV-derived T_m_ values revealed this stability trend for the duplex domain: **cycNU172-EG2** ≈ **cycNU172-EG3** ≈ **cycNU172-Pro** > **cycNU172-Ph** > NU172 in the K^+^-rich buffer, and **cycNU172-EG3** > **cycNU172-Pro** > **cycNU172-Ph** > **cycNU172-EG2** > NU172 in the Na^+^-rich buffer solution (Figure S8 and Table S2), suggesting a marked stabilization of these structural motifs in all the cyclic derivatives, not strictly dependent on the nature and length of the linker.

Interestingly, the apparent T_m_ values obtained by monitoring the 260 and 295 nm melting curves were always significantly different, according to the bimodular nature of the duplex/quadruplex aptamers (Table S2). Furthermore, influence of the nature of the cation in the analyzed buffer solution proved to be very modest on the thermal stability of the duplex domains [71], differently from what observed for the G4 ones [62]. Thus, monitoring the UV signal at 260 nm, both unmodified NU172 and its cyclic analogues showed approximately the same behavior with respect to the saline conditions used. The only exception to this trend was **cycNU172-EG2**, which produced sensibly different apparent T_m_ values, i.e., 62 and 71 °C, respectively in K^+^- and Na^+^-rich media, suggesting that in this case the stability of the duplex domain was more strongly influenced by the presence of the G4 module, as also previously found for other anticoagulant bimodular aptamers [49].

#### 2.3.2. CD Spectroscopy Analysis: CD Spectra and CD Thermal Denaturation/Renaturation Measurements

**Figure 3:**
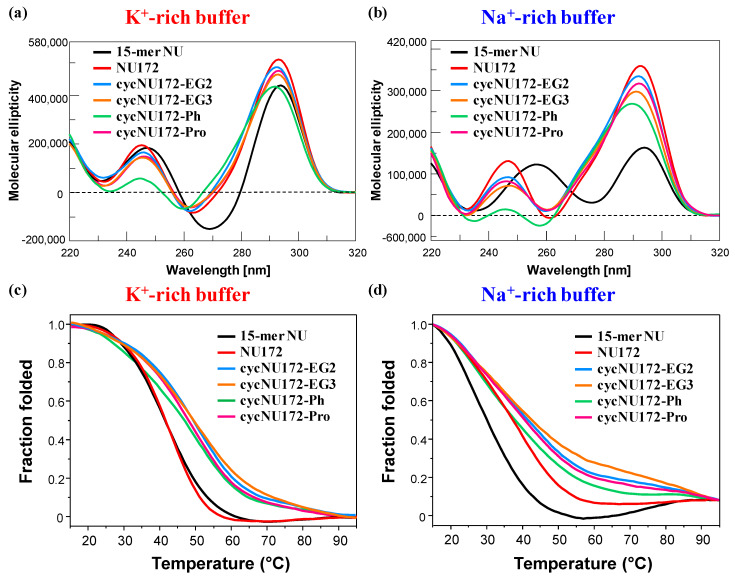
Representative overlapped CD spectra (**a**,**b**) and CD-melting profiles (**c**,**d**) of the cyclic NU172 derivatives (light blue, orange, green, and magenta lines, respectively for **cycNU172-EG2**, **cycNU172-EG3**, **cycNU172-Ph** and **cycNU172-Pro**), recorded at 2 µM concentration and 15 °C in both the selected K^+^- (**a**,**c**) and Na^+^-rich (**b**,**d**) buffer solutions, in comparison with unmodified NU172 and NU (dark red and black lines, respectively). CD-melting profiles—recorded at the maximum ellipticity observed for each oligonucleotide system, using a scan rate of 1 °C/min—are reported as folded fraction of each oligonucleotide system as a function of temperature.

CD spectra and CD thermal denaturation measurements were recorded for the cyclic NU172 analogues in comparison with unmodified NU172 and NU. The CD spectra—recorded at 15 °C and 2 µM oligonucleotide concentration—are reported in Figure 3, respectively in panels a and b for the K^+^- and Na^+^-rich buffers, and their features are summarized in Table S3.

In the presence of K^+^ ions, the CD spectra of NU (Figure 3a, black line) showed spectral properties fully consistent with an antiparallel G4 folding [65,72,73,74,75,76,77,78,79], with two positive signals centered at 294 and 248 nm and one negative CD band with a minimum at 269 nm (Table S3), according to previous findings in similar buffer conditions [52]. On the contrary, in the Na^+^-containing solution, NU produced a CD spectrum (Figure 3b, black line) with two positive maxima at 294 and 257 nm and one negative band with minimum at 276 nm (Table S3). These spectral features—in accordance with previous findings in similar saline solutions [50]—suggested the coexistence in solution of multiple conformations, with a predominance of antiparallel G4 folding. Thus, in order to get a deeper insight into the conformational behavior of NU in solution, the recorded CD spectra were further elaborated by a singular value decomposition (SVD) analysis [79] This analysis revealed that, in both the analyzed saline conditions, the antiparallel G4 conformation prevailed (ca. 83 and 63%, respectively for K^+^- and Na^+^-rich buffers, Table S4), accompanied in the K^+^-rich solution by a small amount of hybrid G4 topology (ca. 17%), and in the presence of Na^+^ ions by a consistent percentage of parallel G4 foldings (ca. 36%).

As far as NU172 was concerned (Figure 3, dark red line), in both the explored saline conditions, this duplex/quadruplex aptamer displayed two positive maxima, around 293 and 245 nm, and one negative band centered at 262 nm (Table S3), clearly indicating the formation of an antiparallel G4 structure, according to the literature [49,50,52]. Thus, also in the Na^+^-rich buffer, the duplex module of NU172 strongly shifted the equilibrium of its G4 core towards the antiparallel G4 topology, acting as a conformational constraint motif for the G4-forming NU sequence.

Notably, in both saline solutions the superimposition of the CD spectra of NU172 and NU underlined for the mixed duplex/quadruplex a marked variability in both position and intensity of the minimum at 260 nm, which can be reasonably attributed to the duplex domain (Table S3). In particular, in the K^+^-rich buffer, the difference spectrum between NU172 and NU—both sharing the same antiparallel G4 motif—produced one positive and one negative CD signal at ca. 280 and 260 nm, respectively, consistent with the GC base pairs contribution (Figure S15) [74].

Then, in both saline conditions, CD spectra of **cycNU172-EG2**, **cycNU172-EG3** and **cycNU172-Pro** (Figure 3a,b, light blue, orange, and magenta lines, respectively) were essentially superimposable to that of unmodified NU172, showing similar amplitudes of the main CD bands, consisting in two positive bands around 291–293 and 245–247 nm, and one negative band with minima at 260–263 nm (Table S3). Only in the case of **cycNU172-Ph**, sensible changes in the spectral properties were observed in both the phosphate buffer solutions, with a marked reduction of the overall CD band intensity (above all for the positive band at ca. 240 nm) and slight shifts in both the minima and maxima (Figure 3a,b, green line and Table S3). These differences suggested slight conformational differences in the G4 arrangement in solution, however, they maintained the prevalent antiparallel G4 folding, and could be also associated with additional stacking interactions due to the presence of an aromatic ring in the linker of **cycNU172-Ph**.

However, for all the investigated oligonucleotides, higher molar ellipticities were observed in K^+^ than in Na^+^-containing media, indicating a higher structuration degree in the first case, according to the well-known ability of K^+^ to stabilize G4 structures better than Na^+^ [23,78,80,81,82,83].

Taken together, the collected CD data confirmed the ability of the cyclic NU172 variants to form antiparallel G-quadruplex structures, further confirming that their cyclic nature did not impair the G4 structuring ability of the selected oligonucleotide nor affected its folding topology, as also previously evidenced in the case of the cyclic TBA analogues [32,33].

Differences in the behavior of the cyclic NU172 derivatives were observed by comparing their CD-melting profiles, obtained monitoring the CD signal—at the maximum ellipticity for each oligonucleotide system, according to the values reported in Table S3—on varying the temperature. For these experiments, all the cyclic oligonucleotides were analyzed at 2 µM concentration in both the K^+^- and Na^+^-rich buffer solutions in the 15–95 °C range, in comparison with unmodified NU172 and NU. In Figure 3 (panels c and d) the overlapped CD-melting profiles of all the studied oligonucleotides are reported in terms of folded fraction as a function of the temperature, while in Figures S9–S14 the overlapped CD-melting/annealing profiles for each compound are shown, along with the superimposition of the spectra recorded at 5 °C steps during each heating and cooling experiment. An overview of the apparent T_m_ values derived by CD-monitored thermal denaturation/renaturation measurements is reported in Table S5.

In the presence of K^+^ ions, NU and NU172 (Figure 3c, Figures S16a and S17a) showed similar CD-melting profiles with sigmoidal curves and apparent T_m_ values around 40 °C (Table S5), in accordance with those found in the UV-monitored thermal experiments. In the selected Na^+^-rich solution, NU172 showed an apparent T_m_ value of 36 °C, consistently with previous investigations in similar saline conditions [49,50], also indicating an enhanced thermal stability with respect to NU (Figure 3d, Figures S16b, S17b and Table S5). Thus, in accordance with the UV-monitored thermal experiments, in the presence of K^+^ ions, NU172 and NU unfolded approximately at the same temperature, while in the Na^+^-rich solution NU172 was markedly more stable than the “nude” G4 domain NU, as a consequence of stabilization effects on the aptamer folding provided by the duplex module.

As far as the cyclic variants of NU172 are concerned, all the derivatives revealed sigmoidal profiles with notably increased thermal stability compared to the linear oligonucleotide (Figure 3 and Figures S18–S21 and Table S5). Only **cycNU172-Ph** (Figure 3d and Figure S20b) exhibited in the Na^+^-rich solution negligible differences compared to the linear aptamer, showing very similar T_m_ values within the experimental error (Table S5).

Taken together, the CD-derived T_m_ values revealed this trend of G4 stability: **cycNU172-EG2** ≈ **cycNU172-EG3** > **cycNU172-Pro** > **cycNU172-Ph** > NU172 > NU in the K^+^-rich buffer, and **cycNU172-EG2** ≈ **cycNU172-EG3** > **cycNU172-Pro** > **cycNU172-Ph** ≈ NU172 > NU in the Na^+^-rich buffer solution (Table S5), with data in accordance with the general finding that G4 structures are more stable in K^+^- than in Na^+^-rich solutions [23,78,80,81,82,83]. In all cases, a nice sigmoidal behavior was observed (Figures S16–S21), with no significant hysteresis on comparing the heating and cooling profiles, thereby indicating that, under the experimental conditions used, the related denaturation/renaturation processes were fully reversible (Figures S16–S21 and Table S5).

CD spectra acquired in 5 °C steps during each heating and cooling experiment (Figures S16–S21) showed for all the oligonucleotides here analyzed a progressive reduction of the CD signals on increasing the temperature. Notably, spectra of unfolded forms of NU172 in both the selected buffer solutions still showed a positive peak around 280 nm, contrarily to NU. All the cyclic NU172 variants, in both saline conditions, behaved as their natural counterpart from a qualitative point of view, with full recovery of the initial spectral features after a melting/annealing cycle (Figures S16–S21).

Interestingly, the superimposition of the spectra acquired during the heating/cooling measurements (Figures S16–S21) denoted for NU, NU172 and its cyclic variants the presence of an isodichroic point at about 280 nm—indicative of a two-state process [53,84]—only in the investigated K^+^-rich buffer. In contrast, no isodichroic point was present for all the analyzed oligonucleotides in the Na^+^-rich medium, suggesting that in these saline conditions they did not unfold following a two-state process, but rather underwent sequential melting of each domain (for bimodular aptamers) or multi-step conformational changes (for NU).

Therefore, only the CD spectral data collected in the selected K^+^-containing solution could be elaborated through van’t Hoff analysis, which involves a “two-state process” assumption allowing for the determination of the thermodynamic parameters [64,85,86]. The thermodynamic change values (ΔH^0^ and ΔS^0^) were calculated from the obtained CD curves by the Marquardt nonlinear least-squares method used by Petersheim and Turner [87] adapted to a monomolecular system. The ΔH^0^ and ΔS^0^ values derived from fittings and the calculated ΔG^0^ values at 298 K are reported in Table S6. Both the standard enthalpy and entropy change values observed for NU172 were sensibly higher in absolute values than those found for NU, consistently with the stabilization role played by the duplex motif. Moreover, compared to NU, NU172 also showed higher ΔS^0^ change values for the unfolding process, suggesting a more rigid structure due to the presence of the duplex domain. Taken together, the standard enthalpy and entropy change values found for NU172 confirmed that the duplex module dramatically enhanced the thermodynamic stability of its G4 domain.

In the case of the cyclic NU172 variants, analogously to their linear counterpart, aptamer structuring appeared as an enthalpy-driven process; in particular, the ΔH^0^ values were consistent with the unfolding of two G-tetrads of the G4 structure [88,89,90,91]. However, all these cyclic analogues showed standard enthalpy and entropy changes lower in absolute values compared to NU172. Overall differences in the enthalpic (ΔH^0^) and entropic (-TΔS^0^) terms for the cyclic aptamers were generally compensated. Where observed, the increased stability of the G4 structure of the cyclic analogues with respect to unmodified NU172 could be mainly attributed to a favorable ΔS^0^ change which balanced the decrease of the ΔH^0^ term. The entropic changes were definitively in accordance with the expected structural preorganization of the cyclic NU172 analogues, also previously observed for the cyclic TBA variants investigated by our group [32,33].

### 2.4. Non-Denaturing Polyacrylamide Gel Electrophoresis

**Figure 4:**
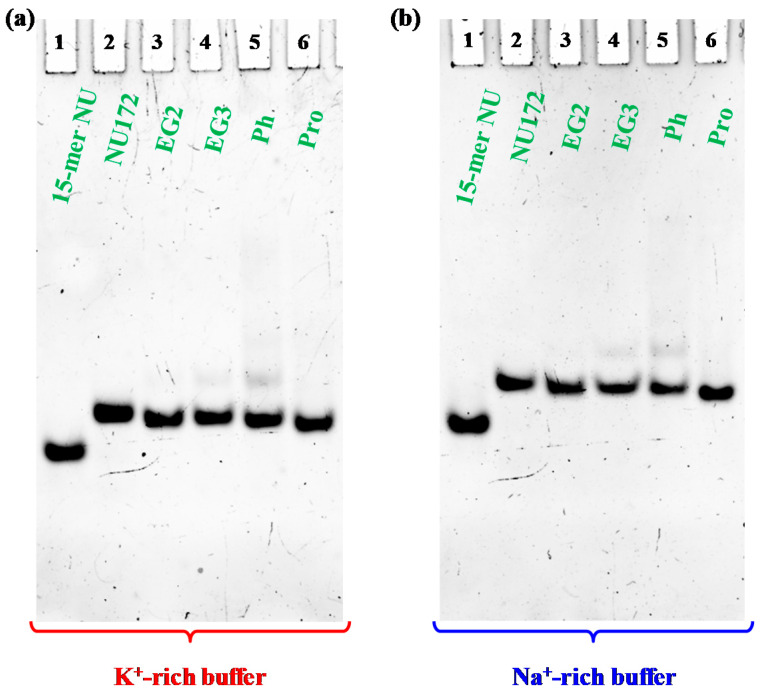
Representative 15% polyacrylamide gel electrophoresis under native conditions of the oligonucleotide samples at 1 μM concentration (only NU was analyzed at 2 μM conc.) in the selected K^+^- (**a**) and Na^+^-rich (**b**) buffer solutions, run at 80 V at room temperature (r.t.) for 2 h in Tris-Borate-EDTA (TBE) 1X buffer; lane 1: NU; lane 2: NU172; lane 3: **cycNU172-EG2**; lane 4: **cycNU172-EG3**; lane 5: **cycNU172-Ph**; lane 6: **cycNU172-Pro**.

In order to confirm the monomolecular structure of the cyclic NU172 analogues and the absence of oligomers or additional superstructures in solution [92], a non-denaturing PAGE analysis was carried out. In Figure 4, a representative example of a native 15% polyacrylamide gel is reported.

Electrophoretic analysis revealed that, in both phosphate buffers, each cyclic NU172 analogue migrated as a single band on the gel, indicating the presence of a single species, with a slightly increased electrophoretic mobility with respect to unmodified NU172 (Figure 4).

Considering that G4 structures migrate on the gel on the basis of their conformation and compactness and that the charge/mass ratio of the cyclic NU172 analogues is very similar to that of the linear aptamer, the PAGE results suggested a more compact structure for all the cyclic oligonucleotides investigated compared to NU172, allowing for a higher mobility through the gel. Since this behavior was already observed for the previously described cyclic TBAs [32,33], the increased mobility could be directly ascribed to the cyclic structure of these derivatives, even if in the case of the smaller TBA analogues the effect of cyclization on their migration ability was more pronounced [32,33].

A small amount of an additional retarded band was observed only in the case of **cycNU172-Ph**, on the gel in both the explored saline conditions (Figure 4, lanes 5), suggesting the presence of another conformation—different from the predominant one—in accordance with CD spectra results. Under the same conditions, NU (Figure 4, lanes 1) migrated faster than NU172 and its cyclic variants, in full agreement with its smaller size (15 nucleotides vs. 26).

In all cases, bands with lower mobility, corresponding to aggregate or higher order G4 species, were not found, confirming the unique presence of monomolecular G4 structures, similarly to the case of native NU172, as also previously demonstrated [52].

### 2.5. Nucleases Stability Assay

One of the main limitations to the use of oligonucleotides in vivo is their susceptibility to nuclease degradation in serum, leading to inactive small DNA fragments, rapidly eliminated from the systemic circulation. Therefore, the integrity of a selected oligonucleotide in serum is one of the most crucial parameters for the evaluation of its potential in vivo use.

**Figure 5:**
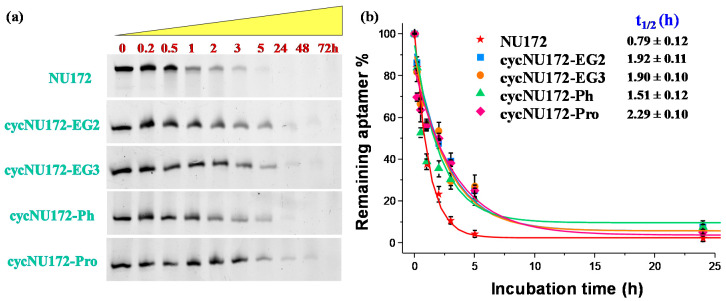
Enzymatic resistance experiments performed on NU172 and its cyclic derivatives incubated in 80% fetal bovine serum (FBS) as monitored by 20% denaturing polyacrylamide gel electrophoresis up to 72 h (time points: 0, 0.2, 0.5, 1, 2, 3, 5, 24, 48 and 72 h): (**a**) representative 20% denaturing PAGE (8 M urea) at 1 μM sample concentration, run at constant 200 V at r.t. for 2.5 h in TBE 1X as running buffer; (**b**) intensity of each oligonucleotide band on the gel expressed as percentage of the remaining intact aptamer (see legend for detail) with respect to the untreated oligonucleotide, analyzed up to 24 h. Data are reported as mean values ± SD (error bars) for multiple determinations (at least five). Obtained values were also fitted with an equation for first order kinetics (lines), allowing calculation of the half-life in serum of each aptamer (t_1/2_).

In order to determine their stability in serum, and in particular, verify if the inserted circularizing linkers were able to retard the nuclease degradation, the novel cyclic NU172 variants were tested in their resistance against enzymatic digestion. Briefly, all the cyclic NU172 derivatives—along with unmodified NU172 used as reference—were incubated in 80% (*v/v*) fetal bovine serum (FBS) at 37 °C and monitored for 72 h. At fixed times, aliquots of these mixtures were collected, supplemented with formamide to immediately stop the enzymatic degradation and then analyzed by 20% denaturing PAGE (Figure 5a). The intensity of each oligonucleotide band on the gel was then calculated and normalized with respect to that of untreated oligonucleotide. Subsequently, the fitting of the experimental points allowed for the calculation of the half-life (t_1/2_) values, defined as the time at which the amount of a selected sample decayed by 50%. The percentages of each remaining aptamer along with the corresponding fitting curves are presented in Figure 5b for a comparative view of the nuclease digestion results related to the first 24 h monitoring, and separately reported for each system up to 72 h in Figure S22.

For unmodified NU172, PAGE experiments revealed a progressive reduction of its band intensity in the time range from 1 to 3 h, and its complete disappearance within 5 h (Figure 5a) with a half-life of 0.79 h (Figure 5b). Conversely, after 5 h treatment, all the cyclic NU172 derivatives were still detectable, at least for 20–27% of their initial amount, suggesting a consistent nuclease degradation after this incubation period, as indicated by the progressive disappearance of the corresponding bands. Even if each cyclic derivative showed a peculiar behavior in the first 5 h of monitoring, all the cyclic NU172 analogues were completely degraded within 24 h (Figure 5a).

Taken together, the PAGE analysis clearly indicated that all the cyclic NU172 variants had from 1.9 to 2.9-fold enhanced t_1/2_ values with respect to unmodified NU172 (Figure 5 and Figure S22). In particular, the nuclease stability was found to be slightly dependent on both the nature of the circularizing linker and its length, with t_1/2_ values found between 1.5 h and 2.3 h. The lowest nuclease stability was found for **cycNU172-Ph**, with a t_1/2_ of 1.51 h, while **cycNU172-EG2** and **cycNU172-EG3** showed a similar behavior, with t_1/2_ values around 1.90 h. In turn, **cycNU172-Pro** proved to be the most stable derivative against nuclease degradation, with t_1/2_ of 2.29 h, i.e., almost three-fold enhanced compared to NU172. The general trend of serum nuclease resistance was therefore: **cycNU172-Pro** > **cycNU172-EG2** ≈ **cycNU172-EG3** > **cycNU172-Ph** > NU172.

These data comprehensively confirmed that end-capped oligonucleotides—as obtained by covalently linking their 5′ and 3′-ends—were indeed less accessible to the nuclease attack than their linear counterpart. Thus, cyclization of an oligonucleotide may provide a convenient and precious strategy to effectively reduce its exonuclease susceptibility, also improving the serum stability of G-quadruplex structures in comparison to similar species with solvent-exposed extremities, as already proved for the previously developed cyclic TBA derivatives [32,33].

### 2.6. Coagulation Experiments

The anticoagulant activity of the cyclic NU172 derivatives, with respect to their linear analogue and NU, was evaluated by following the thrombin-catalyzed conversion of fibrinogen to fibrin by means of light scattering (LS) experiments [93]. Indeed, a marked increase of the scattered light intensity can be detected upon conversion of fibrinogen to fibrin, as a result of the dependence of the scattering intensity on the size of the objects in solution. Thus, normalized LS intensity curves, as a function of time, measure the progression of fibrin formation and thus allow monitoring the coagulation process.

**Figure 6:**
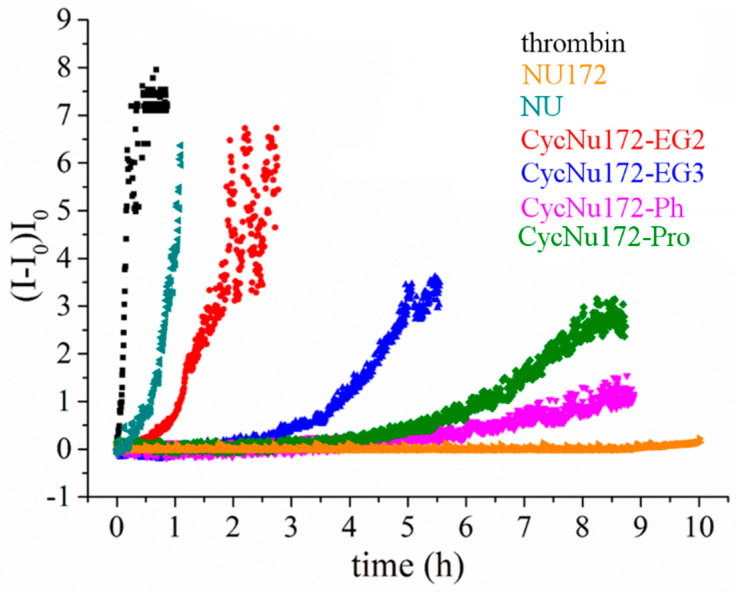
Coagulation curves of fibrinogen in the presence of thrombin and different anticoagulant agents (NU172, NU and cyclic NU172 analogues, see inset for details), evaluated by means of light scattering experiments in phosphate buffer solution (PBS). All the aptamers were analyzed at a thrombin:aptamer ratio of 1:2.

These experiments were performed in PBS (Na^+^-rich buffer), which has an ion content similar to the blood salt composition and is therefore suitable to investigate the anticoagulant activity of these aptamers as potential thrombin inhibitors. The overall results obtained both in the absence and presence of the oligonucleotides at 1:2 thrombin:aptamer molar ratio are reported in Figure 6. In the presence of thrombin, a quick and very steep increase of normalized scattered intensity was observed, indicating a rapid conversion of fibrinogen into larger fibrin strands. Conversely, in the presence of the aptamers, the scattered intensity increased to a lesser extent and more slowly than in the presence of thrombin alone. However, the slope of the curves and the final value of intensity reached were significantly different for each investigated aptamer. In particular, in the case of NU172, an almost flat coagulation curve was observed, whereas the coagulation curve in the presence of NU was not very different from that recorded for thrombin alone.

Therefore, the analysis of the coagulation curves clearly highlighted a very distinct anticoagulant ability for the different investigated aptamers. In particular, NU172 appeared as the most active and NU as the least effective inhibitor in the explored series, whereas the investigated cyclic NU172 derivatives exhibited an intermediate behavior among these two limit cases. In detail, in the presence of **cycNU172-Ph** and **cycNU172-Pro** a long lag time along with a lower slope of the curve were observed with respect to **cycNU172-EG2** and **cycNU172-EG3**, suggesting a higher inhibitory activity for the cyclic molecules obtained via the oxime ligation method.

Aiming at comparing the inhibition properties of all the cyclic NU172 derivatives in a quantitative way, their anticoagulant activity was calculated as the ratio between the initial slope of the normalized scattered intensities curves in the absence and presence of the tested oligonucleotides, thus providing an estimation of the coagulation rate (Table 2).

All the cyclic NU172 derivatives were less effective than NU172, but significantly more active than NU, proving to have comparable anticoagulant activity as in TBA [52]. Even the least effective among the cyclic aptamers in this series, i.e., **cycNU172-EG2**, was five-fold more active than NU; in turn, **cycNU172-EG3** and **cycNU172-Pro** were even more potent, with anticoagulant activities respectively 15- and 25-fold higher than NU. Interestingly, upon comparing **cycNU172-EG2** and **cycNU172-EG3**, the extension of the linker from 24 to 27 atoms led to a significant increase of the inhibitory activity, suggesting that some higher flexibility is required to obtain a better thrombin inhibition ability. Finally, the most effective cyclic analogue was found to be **cycNU172-Ph**, with an anticoagulant potency 100-fold higher than NU. Compared to its linear precursor, this analogue proved to be four times less effective, but showed a strong bioactivity, in the same high order of magnitude as NU172, which is one of the strongest anticoagulant aptamers known thus far.

## 3. Materials and Methods

### 3.1. General Methods

All the reagents and solvents were of the highest commercially available quality and were used as received. Nuclease-free water, acrylamide/bis-acrylamide (19:1) 40% solution, GelGreen Nucleic Acid Stain, 6X Orange DNA Loading Dye and Tris-Borate-EDTA (TBE) 10X were purchased from VWR. Formamide, urea, ammonium persulfate (APS) and tetramethylethylenediamine (TEMED) were purchased from Sigma Aldrich. Fetal Bovine Serum (FBS) was provided by Euroclone.

Among the oligonucleotides here studied, unmodified NU172 and NU were purchased from biomers.net GmbH (Germany) as HPLC-purified oligomers. Their quality was checked by HPLC and MALDI-TOF MS by the commercial suppliers. The cyclic NU172 analogues were synthesized and purified as described below. The purity of all the oligonucleotides was further confirmed by denaturing 20% PAGE analysis.

### 3.2. Synthetic Procedures

#### 3.2.1. Synthesis of the Cyclic NU172 Derivatives

The NU172 sequence was elongated from the azido-derivatized support **1** or ethyleneglycol oxime solid support **8** [59] on an ABI 394 DNA synthesizer, with a 1 μmol scale cycle for 3 times, according to standard phosphoramidite chemistry protocols. The detritylation step was performed for 65 s using 3% TCA in CH_2_Cl_2_. For the coupling step, benzylmercaptotetrazole (0.3 M in anhydrous CH_3_CN) was used as the activator with commercially available 2′-deoxynucleoside 2-cyanoethyl, *N*,*N*-diisopropylphosphoramidites with regular protecting group or fast-deprotection ones (*N^2^*-*t*BuPac-dG, *N^6^*-Pac-dA or *N^4^*-Ac-dC) (0.075 M in CH_3_CN, 30 s coupling time), or di-ethyleneglycol propargyl phosphoramidite **2**, triethyleneglycol propargyl phosphoramidite **3**, MMTrN-EG_3_ phosphoramidite **11** (0.1 M in CH_3_CN, 60 s coupling time). Commercially available solutions of phenoxyacetic or acetic anhydride were used for the capping step (Cap A: anhydride:pyridine:THF 10:10:80 *v/v/v*) combined with Cap B solution (10% *N*-methylimidazole in THF) for 60 s or 10 s, respectively. An iodine solution (0.1 M I_2_, THF:pyridine:water 90:5:5, *v/v/v*) was applied for 15 s for the oxidation step.

#### 3.2.2. Cyclization by CuAAC (cycNU172-EG2 and cycNU172-EG3)

Oligonucleotide-functionalized Controlled Pore Glass (CPG) beads **4** and **5** were introduced into a sealed vial and treated with concentrated aqueous ammonia (2 mL) overnight at 40 °C. The supernatant was withdrawn and evaporated. The linear modified-NU172 oligonucleotides **6** and **7** were dissolved in water and then analyzed by UV, HPLC and MALDI-TOF MS. The amount of crude **6** and **7**, determined by UV measurements at 260 nm, was 1.21 and 1.46 µmol, respectively. Analytical RP-HPLC retention time of 14.71 min for **6** and 15.00 min for **7** were found.

Freshly prepared aq. solutions of CuSO_4_ (5 eq, 100 mM) and sodium ascorbate (10 eq, 500 mM) were added to a solution of linear NU172 **6** (1.21 µmol) or **7** (1.46 µmol) dissolved in 1 mL of H_2_O and KCl (50 mM, 4 mL). The vials containing the resulting mixtures were sealed and stirred at room temperature (r.t.). Then, HPLC monitoring at 3.5 h showed almost complete cyclization, with only tiny amounts of the starting linear oligonucleotides. After 4 h, the mixtures were desalted by size exclusion chromatography (SEC) on a NAP-25 column (GE-Healthcare), producing crude cyclic compounds which were frozen and lyophilized. The crudes were then purified by HPLC affording **cycNU172-EG2** (160 nmol, 13% overall yields, analytical RP-HPLC retention time: 12.09 min, MALDI-TOF MS: *m*/*z*: [M-H]^−^ = calc. 8667.70, found 8666.90) and **cycNU172-EG3** (303 nmol, 22% overall yields, analytical RP-HPLC retention time: 12.42 min, MALDI-TOF MS: *m*/*z*: [M-H]^−^ = calc. 8711.75, found 8711.56) which were frozen and lyophilized.

#### 3.2.3. Cyclization by Bis-Oxime Ligation (cycNU172-Ph and cycNU172-Pro)

Solid-supported oligonucleotide **10** (2.5 µmol), synthesized using fast-deprotection protecting groups, was treated with a concentrated ammonia solution (4 mL) at r.t. for 3 h. After washing with water and methanol, the CPG beads were transferred into a vial and treated with a 50 mM CH_3_ONH_2_ HCl solution in 0.4 M NH_4_Ac buffer, pH = 4.2 (2 mL) for 6 h at r.t. The linear NU172 **11** was desalted by SEC cartridges (NAP-10), recovering 464 nmol of the target compound which were then lyophilized. Analytical RP-HPLC retention time: 11.62 min. MALDI-TOF MS: *m*/*z*: [M-H]^−^ = calc. 8520.47, found 8522.61.

The crude **11** was dissolved in water (450 nmol in 450 µL) and to this solution, 50 mM KCl (300 µL), 0.4 M NH_4_Ac buffer pH = 4.2 (3.0 mL) and 1.2 eq of bis-aldehyde (terephthalaldehyde or glutaraldehyde 2.5 mM in MeOH) were sequentially added. After 3 h at r.t., the crude was desalted by SEC cartridges (NAP-25) and purified by reverse phase C18 HPLC, affording the target cyclic derivatives. **cycNU172-Ph**: 29 nmol starting from 200 nmol of **11**, 15% yield. Analytical RP-HPLC retention time: 12.91 and 13.14 min for E/Z isomers, MALDI-TOF MS: *m*/*z*: [M-H]^−^ = calc. 8618.58, found 8619.08. **cycNU172-Pro**: 42 nmol starting from 250 nmol of **11**, 17% yield. Analytical RP-HPLC retention time: 12.96 min, MALDI-TOF MS: *m*/*z*: [M-H]^−^ = calc. 8584.56, found 8584.17.

### 3.3. Preparation of the Oligonucleotide Samples

Purified and lyophilized oligonucleotides were dissolved in a defined volume of nuclease-free water. Their concentration was determined by UV measurements on a JASCO V-630 UV-vis spectrophotometer equipped with a Peltier Thermostat JASCO ETCS-761, by using a quartz cuvette with a 1 cm path length (1 mL internal volume, Hellma), recording the absorbance at 260 nm and 90 °C. The molar extinction coefficient of 277.162 cm^−1^ M^−1^ was used for both NU172 and its cyclic derivatives, as calculated for the unstacked oligonucleotides using Oligo Calculator [94], assuming for the latter ones that the inserted circularizing linkers did not provide any contribution to the UV absorbance at 260 nm. In turn, the molar extinction coefficient of 168.890 cm^−1^ M^−1^ was used for NU.

The UV spectra were recorded in the range 220–320 nm with a medium response, a scanning speed of 100 nm/min and a 2.0 nm bandwidth with the appropriate baseline subtracted. Taking a suitable aliquot from the initial stock solutions in H_2_O, all the investigated oligonucleotides were then diluted in the selected K^+^- (10 mM KH_2_PO_4_/K_2_HPO_4_, 70 mM KCl, 0.2 mM EDTA, pH = 7.3) or Na^+^-rich (PBS: 137 mM NaCl, 2.7 mM KCl, 10 mM NaH_2_PO_4_/Na_2_HPO_4_, 1.8 mM KH_2_PO_4_/K_2_HPO_4_ pH = 7.3) phosphate buffer solutions. Thereafter, the samples were annealed by heating the solutions for 5 min at 95 °C and then left to slowly cool to r.t. overnight, as to allow their structuring into the thermodynamically most stable conformations [95]. The annealed samples were eventually kept at 4 °C until their subsequent use.

### 3.4. UV Spectroscopy

UV spectra and UV-thermal denaturation/renaturation measurements were performed on a JASCO V-630 UV-vis spectrophotometer equipped with a Peltier Thermostat JASCO ETCS-761, by using a quartz cuvette with a 1 cm path length (1 mL internal volume, Hellma). All the investigated oligonucleotides were dissolved in the selected phosphate buffer solution so to obtain 2 μM solutions, which were then slowly annealed.

In particular, the UV spectra—in the range 220–320 nm—were recorded at 15 and 90 °C using the proper baseline subtraction (scanning speed of 100 nm/min) [60,64]. Thermal difference spectra (TDS) were then mathematically obtained by subtracting the absorbance spectrum obtained at 15 °C (i.e., a temperature below the T_m_, at which the oligonucleotide is fully structured) from the one recorded at 90 °C (corresponding to a temperature above the T_m_, at which the aptamer G4 structure is fully denatured) [63,64,66]. UV measurements at each temperature were repeated twice.

In order to facilitate the comparison of the spectral data, all the obtained differential spectra were then normalized to the maximum of absorbance simply by dividing the raw data in the 220–320 range by its maximum value, so that the highest positive peak gets a Y-value of +1 [63]. From normalized spectra, TDS factors (ΔA_240_/ΔA_295_, ΔA_255_/ΔA_295_ and ΔA_275_/ΔA_295_) were also calculated in both the analyzed saline conditions as the ratios between the absolute absorbance values at different wavelengths. TDS factor evaluation was possible only for NU being a single G4 structure, while in the case of the bimodular duplex/quadruplex structures the analysis of the absorbance ratio did not provide consistent results [65].

The absorbance vs. temperature profiles of the target oligonucleotides were monitored following the absorbance changes (at 295 or 260 nm, as specified) in the temperature range 15–90 °C with a scan rate of 1 °C/min [64,69]. UV melting profiles at both 260 nm and 295 nm were reported as normalized ΔA values (NΔA) as a function of temperature. Normalized ΔA was calculated as: NΔA = [(Abs_obs_ (T) − Abs_min_)/(Abs_max_ − Abs_min_)], where A_obs_ is the UV absorbance at 260 or 295 nm at each temperature, Abs_max_ and Abs_min_ are respectively the highest and the lowest absorbance values recorded during the thermal experiment. The apparent melting temperatures (T_m_) values were determined from the normalized data as the temperature at which half of the sample is folded and half is unfolded, i.e., NΔA = 0.5 (associated error: ±1 °C). Each experiment was performed in duplicate.

### 3.5. Circular Dichroism (CD) Spectroscopy

CD spectra and CD-monitored denaturation/renaturation experiments were recorded—using a quartz cuvette from Hellma (1 cm path length and 3 mL internal volume)—on a Jasco J-715 spectropolarimeter supplied with a Peltier-type temperature control system (model PTC-348WI). The following CD parameters were used: 220–320 nm range, data pitch 1 nm, band width 2 nm, response 4 s, scanning speed 100 nm/min, 3 accumulations, with the proper baseline subtraction [78]. All the investigated oligonucleotides were dissolved in the selected saline conditions at 2 μM concentration.

Thermal melting/annealing curves were recorded following the CD signal intensity—at the maximum ellipticity—as a function of the temperature with a scan rate of 1 °C/min. In parallel, CD spectra in 220–320 nm spectral window and 5 °C steps were also recorded in the 15–95 °C temperature range for both the selected buffer solutions. Each experiment was carried out in triplicate.

The molar ellipticity [θ] (deg cm^2^ dmol^−1^) was then calculated using the equation [θ] = θ_obs_/10 × *l* × *C*, where θ_obs_ is the recorded ellipticity (in mdeg), *C* is the oligonucleotide molar concentration used in the experiment, and *l* is the optical path length of the cell (expressed in cm).

For the singular value decomposition (SVD) analysis—performed only on NU presenting a single G4 domain—CD spectra were also normalized to molar circular dichroism, Δε (M^−1^·cm^−1^) = θ/(32980 × *C* × *l*), where θ is the CD ellipticity in millidegrees, *C* is DNA concentration in mol L^−1^, and *l* is the path length in cm. Then, the obtained spectra were analyzed using the advanced software developed by del Villar-Guerra et al. [79].

Data were also converted into folded fraction (α) calculated as: α = [θ_obs_(T) − θ_U_)/(θ_F_ − θ_U_)], where θ_obs_(T) is the molar ellipticity—at the maximum observed for each oligonucleotide—for each temperature, while θ_F_ and θ_U_ are the molar ellipticity values for the folded (T = 15 °C) and unfolded (T = 95 °C) oligonucleotide, respectively. The T_m_ values were calculated from normalized data as the temperature at which half of the sample is folded and half is unfolded, i.e., α = 0.5 (associated error: ±1 °C). The ΔT_m_ values were calculated by subtracting the measured T_m_ value of unmodified NU172 from that observed for each cyclic analogue.

The van’t Hoff analysis was only possible for CD-monitored unfolding experiments performed in the selected K^+^-rich buffer. The thermodynamic change values (ΔH^0^ and ΔS^0^) were calculated using the method developed by Petersheim and Turner [87] adapted to a monomolecular system, [85] under the assumption ΔCp = 0. The Marquardt nonlinear least-squares method was used to fit the melting curves with the following equation:

ε(T) = A(T)/(*l* × Ct) = αε_f_ + (1 − α) ε_uf_ (1)

where ε(T) and A(T) are respectively the extinction coefficient and the molar ellipticity of the solution respectively at temperature T (K), *l* is the path length, Ct is the total strand concentration. In turn, ε_f_ and ε_uf_ are the extinction coefficients of the folded and unfolded oligonucleotide respectively and are assumed to be linear functions of the temperature:

ε_f_ = a_f_T + b_f_ (2)

ε_uf_ = a_uf_T + b_uf_ (3)

α is the fraction of strands in the folded state and is related to the changes in enthalpy ΔH^0^ and entropy ΔS^0^ according to the equation:

K_eq_ = α/(1 − α) = exp(−ΔH^0^/RT + ΔS^0^/R) (4)

The program fits the experimental melting curve treating ΔH^0^, ΔS^0^, a_f_, b_f_, a_uf_, b_uf_ as variable parameters. In general, R^2^ values better than 0.9999 were obtained. All the thermodynamic parameters are expressed in kJ/mol as mean values ± SD from the multiple determinations.

### 3.6. Polyacrylamide Gel Electrophoresis (PAGE) Analysis

#### 3.6.1. Denaturing PAGE

Denaturing PAGE experiments (8 M urea) were performed according to reported procedures [96], with minor modifications. Briefly, 10 pmol of oligonucleotide samples in water were mixed with formamide (1:2, *v*/*v*) and further heated at 95 °C for 5 min, to completely unfold the samples. Then, all the aptamer mixtures—with the addition of 6× Orange DNA Loading Dye immediately prior gel loading—were analyzed by 20% denaturing PAGE experiments in TBE 1X as running buffer. Gels were run at r.t. and constant 200 V for 2.5 h, then stained with GelGreen Nucleic Acid Stain (0.1 M NaCl enriched) for 30 min and finally visualized with a UV transilluminator (ChemiDoc XRS, BioRad, Milan, Italy). The experiment was carried out in triplicate.

#### 3.6.2. Native PAGE

Slowly annealed oligonucleotides samples (dissolved at 1 μM concentration with the exception of NU, prepared at 2 μM conc.) in both the selected K^+^- and Na^+^-rich buffer solutions were loaded on 15% polyacrylamide gels in TBE 1× as running buffer. All the samples were supplemented with 6× Orange DNA Loading Dye just before loading and then run, under native conditions, at 80 V at r.t. for 2 h. Gels were stained with a GelGreen solution (supplemented with 0.1 M NaCl) for 30 min and finally visualized with a UV transilluminator (BioRad ChemiDoc XRS). Each experiment was performed at least in triplicate.

### 3.7. Enzymatic Stability Assays Monitored by Gel Electrophoresis Analysis

The evaluation of the oligonucleotide stability in serum was performed by gel electrophoresis analysis according to reported procedures [97], with minor modifications. Briefly, all the oligonucleotides—previously annealed in PBS buffer at 17 μM conc.—were incubated in 80% FBS at 37 °C. Then, at fixed times, 3 μL of the samples (corresponding to 10 pmol) were collected, mixed with formamide (1:2, *v/v*) to immediately quench the enzymatic degradation, heated at 95 °C for 5 min, and finally stored at −20 °C until subsequent analysis. Thereafter, all the samples—supplemented with 5% glycerol immediately before loading—were analyzed by electrophoresis on 20% denaturing PAGE using 8 M urea in TBE 1X as running buffer. The gels were run at r.t., at constant 200 V for 2.5 h, then stained with GelGreen Nucleic Acid Stain (supplemented with 0.1 M NaCl) for 30 min and finally visualized with a UV transilluminator (BioRad ChemiDoc XRS). Each experiment was performed at least 5 times. The intensity of the DNA bands on the gel, at each collected time, was then calculated by using the FiJi software and normalized with respect to the initial one (untreated oligonucleotides). Percentages of the remaining intact oligonucleotide are reported as mean values ± SD for the multiple determinations. Half-life times of each oligonucleotide (t_1/2_) was obtained by fitting the values with an equation for first order kinetics.

### 3.8. Anticoagulant Activity by Light Scattering Measurements

Evaluation of the anticoagulant activity of cyclic NU172 analogues, unmodified NU172 and NU as control oligonucleotides, was performed by means of light scattering (LS) experiments, according to reported procedures [93]. LS measurements were carried out with a home-made instrument composed of a Photocor compact goniometer, a SMD 6000 Laser Quantum 50 mW light source operating at 5325 Å, a photomultiplier (PMT-120-OP/B) and a correlator (Flex02−01D) from *Correlator.com*. The intensity was measured at scattering angle of 90° and all the experiments were performed at (25.00 ± 0.05) °C with temperature controlled through the use of a thermostat bath.

A 2.4 μM fibrinogen solution in PBS was placed in a light scattering (LS) cuvette and left to equilibrate in the instrument for 20 min. Then, an equal volume of a 10 nM thrombin solution was added up in order to obtain a final concentration of 1.2 μM for fibrinogen and 5 nM for thrombin. Immediately after thrombin addition, the light scattered intensity was registered every 2 s until it reached a constant value. In the case of the experiments in the presence of the aptamers, these were added to fibrinogen solutions so to have a thrombin:aptamer molar ratio of 1:2. All the experiments were performed at least in triplicate. Coagulation curves were obtained by plotting the normalized scattered intensity *nI* as a function of time according to the equation:

(5) $$I=\frac{I-I_{o}}{I_{o}}$$

where *I* is the scattering intensity registered during the course of experiment and *I_o_* the scattering intensity at the start of the experiment. By linearly fitting the initial increase of *nI* upon the lag time, an estimation of the coagulation rate is obtained. Finally, the anticoagulant activity was calculated as the ratio between the coagulation rate in the presence of thrombin alone and that in the presence of both thrombin and each aptamer.

## 4. Conclusions

NU172 is a 26-mer DNA aptamer consisting of one G-quadruplex and one duplex domain, able to effectively inhibit thrombin activity and clot formation. The crystal structure of the NU172-thrombin complex revealed that the aptamer binds the thrombin exosite I through a highly complementary surface recognition involving all three loops of the G4 module, due to the peculiar structure of the duplex/quadruplex junction involving residues from both the G4 loops and the linkers [52]. Thus, although the duplex domain does not directly interact with thrombin, it is crucial for the correct fold of the G4 loops, optimization of the protein-ligand interaction and inhibition of the protein activity.

In the aptamer research field, in order to improve the properties of a selected oligonucleotide, of particular interest is gathering a large body of structure–activity relationship data also through the development of new derivatives. Thus, we herein analyzed the influence of flexible circularizing linkers, connecting the 3′- and 5′-ends of NU172 sequence, on the folding properties, thermal stability, nuclease resistance as well as biological activity of the aptamer. Indeed, on TBA, we previously explored the cyclization approach, [32,33] providing—after a careful optimization of the length and chemical nature of the linker—a cyclic version, indicated as cycTBA II, exhibiting enhanced anticoagulant activity, along with a dramatically stabilized G4 structure and higher nuclease resistance in serum compared to linear TBA [33]. These encouraging results prompted us to extend the cyclization strategy also to other G4-forming aptamers, encompassing both a duplex and a G4 domain, in order to improve their biophysical and/or biological properties. Therefore, we focused on NU172 with the basic idea to obtain improved NU172 analogues through a good compromise between increasing the conformational and enzymatic stability of its duplex-quadruplex core, and maintaining some structural flexibility, crucial for thrombin recognition.

Four novel derivatives were thus prepared: in detail, **cycNU172-EG2** and **cycNU172-EG3** were obtained using a Cu(I)-catalyzed azide-alkyne cycloaddition approach, while **cycNU172-Ph** and **cycNU172-Pro** were circularized using the oxime ligation method. We therefore tested and compared two different cyclization procedures, providing—in good yields and few synthetic steps—the target cyclic aptamers, featured by connecting linkers with different chemical properties and peculiar structural elements. Then, we investigated the structural and biophysical properties of the cyclic NU172 analogues by spectroscopic and electrophoretic techniques.

UV and CD spectroscopic data revealed that the overall folding topology of the cyclic NU172 variants was unaffected by the cyclization and that all the investigated aptamers folded into duplex-quadruplex structures similar to that of the linear aptamer, with the G4 module always maintaining its peculiar antiparallel G4 conformation. In both the investigated phosphate buffer solutions, the cyclic analogues showed G4 structures with generally improved thermal stability compared to those found for unmodified NU172. In addition, all these cyclic variants proved to be more resistant to nuclease digestion than their natural counterpart, showing **cycNU172-Pro** as the most stable derivative against enzymatic degradation, with a three-fold higher half-life compared to linear NU172. Thus, the analysis of the cyclic NU172 derivatives further confirmed that the cyclization approach can be a convenient strategy to effectively reduce the susceptibility to nucleases and improve both the serum and thermal stability of bimodular aptamers with respect to their linear parent compounds.

The length and chemical nature of the connecting linker also demonstrated to modulate the anticoagulant activity of the cyclic NU172 analogues to a different extent, providing, in all cases, lower inhibitory activity than unmodified NU172, despite being significantly improved compared to the “naked” G4 module NU, that is in turn comparable to that of TBA [52].

As already reported for several TBA derivatives [32,98,99,100,101], the inserted modifications can affect the biological activity in a way that is not tightly connected with structure stability. Thus, also for NU172, the anticoagulant activity is not exclusively dictated by the intrinsic stability of its G4 motif structure, but mainly by its ability to recognize the protein. Moreover, in the case of NU172, the junction nucleotides are crucial in defining the conformation of the G-quadruplex loops and in turn thrombin binding and inhibition, but information on the loops and junctions cannot be directly obtained from spectroscopic characterizations. Thus, one possibility is that the circularizing linkers inserted in the cycNU172 analogues may somehow interact with the nucleotides at the duplex/quadruplex junction, thus also affecting the G4 loop structuring.

Another possibility is that the reduced anticoagulant activity is strictly connected with lower affinity for the protein. Indeed, we previously demonstrated for a cyclic TBA analogue [32] that the reduced anticoagulant activity nicely correlated with a lower affinity for the protein, suggesting that TBA binding to thrombin involved some mutual interactions between the aptamer and the protein. This could be also true for NU172 and the here described cyclic NU172 analogues, also in the light of a very recent study, evaluating hexitol-based NU172 derivatives, which clearly confirmed the tight thrombin affinity-anticoagulant activity relationships [51].

Remarkably, despite the higher conformational heterogeneity found for **cycNU172-Ph**, this analogue proved to be the most active aptamer within the explored series. This could be explained considering this aptamer as a more flexible structure on which thrombin might act as a molecular chaperone [23,52,102], inducing the oligonucleotide to adopt the most effective functional conformations of both the G-quadruplex core and its loops. As an alternative explanation, the lipophilic benzene ring present in the 24 atom-long linker of **cycNU172-Ph** could be involved in intramolecular interactions, finally promoting a more suitable structuring for recognition/inhibition of the protein. Interestingly, **cycNU172-Ph** exhibited the lowest G4 stabilization, as derived by UV and CD studies, and the highest activity among the analyzed cyclic NU172 aptamers. This result seems to suggest that the over-stabilization of the G-quadruplex module can be responsible of the observed decreased anticoagulant activity.

The characterization performed here underlined the importance of the duplex domain, confirming its key role in the stabilization of the antiparallel G4 topology and in the organization of the appropriate folding for thrombin binding. Additionally, our study also highlighted the importance to render the circularizing linker more flexible, elongating it or introducing even more flexible elements.

Taking into account the great biological significance of NU172 as an anticoagulant, our investigations represent an instructive case study on the interplay between several local features concurring to produce novel, promising aptamers, helpful for the design of optimized chemically modified analogues with improved properties.

## Figures and Tables

**Table 1:** Chemical structure and length of the linkers connecting the 3′- and 5′-ends of NU172, providing the cyclic variants here studied, i.e., **cycNU172-EG2**, **cycNU172-EG3**, **cycNU172-Ph** and **cycNU172-Pro**, showing their peculiar structural elements.

	Name	Sequence (5′–3′)	Length
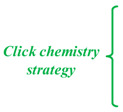	**cycNU172-EG2**	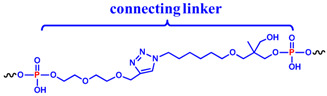	24 atoms
	**cycNU172-EG3**	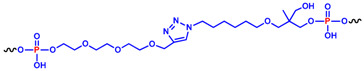	27 atoms
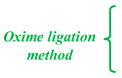	**cycNU172-Ph**	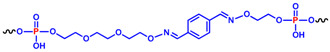	24 atoms
	**cycNU172-Pro**	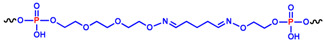	23 atoms

**Table 2:** Anticoagulant activity of different aptamers expressed as the ratio between the coagulation rate in the only presence of the protein and in the presence of both thrombin and each oligonucleotide. The values are reported as the average of at least three independent measurements with their standard deviation.

	Anticoagulant Activity
NU172	900 ± 200
NU	2 ± 0.4
cycNU172-EG2	10 ± 2
cycNU172-EG3	30 ± 6
cycNU172-Ph	200 ± 50
cycNU172-Pro	50 ± 7

## Supplementary Materials

The following are available online at https://www.mdpi.com/1422-0067/21/11/3860/s1, Scheme S1: Synthesis of cycNU172-EG2 and cycNU172-EG3; Figure S1: HPLC profiles of crude oligonucleotides 6 and 7; Figure S2: HPLC and MALDI-TOF profiles of pure cycNU172-EG2; Figure S3: HPLC and MALDI-TOF profiles of pure cycNU172-EG3; Scheme S2: Synthesis of cycNU172-Ph and cycNU172-Pro; Figure S4. HPLC profile of crude oligonucleotide 11; Figure S5. HPLC and MALDI-TOF profiles of pure cycNU172-Ph; Figure S6. HPLC and MALDI-TOF profiles of pure cycNU172-Pro; Figure S7. 20 % denaturing polyacrylamide gel electrophoresis analysis; Table S1. TDS factor analysis for the 15-mer NU; Figure S8. Overlapped UV-melting profiles at 260 nm; Figure S9. UV analysis of the 15-mer NU at 295 nm; Figure S10. UV analysis of NU172 at 295 and 260 nm; Figure S11. UV analysis of cycNU172-EG2 at 295 and 260 nm; Figure S12. UV analysis of cycNU172-EG3 at 295 and 260 nm; Figure S13. UV analysis of cycNU172-Ph at 295 and 260 nm; Figure S14. UV analysis of cycNU172-Pro at 295 and 260 nm; Table S2. Tm values obtained by UV-monitored thermal experiments; Table S3. Specific λ values observed in the CD spectra; Table S4. Prediction of G4 topologies adopted by NU by SVD analysis; Figure S15. CD difference spectrum of NU172 and NU in the K^+^-rich buffer; Figure S16. CD analysis of the 15-mer NU; Figure S17. CD analysis of NU172; Figure S18. CD analysis of cycNU172-EG2; Figure S19. CD analysis of cycNU172-EG3; Figure S20. CD analysis of cycNU172-Ph; Figure S21. CD analysis of cycNU172-Pro; Table S5. Tm values obtained by CD-monitored thermal experiments; Table S6. Standard thermodynamic parameters from van’t Hoff analysis; Figure S22. Nuclease resistance experiments on NU172 and its cyclic derivatives.

## References

[1] Huntington J.A. (2005). Molecular recognition mechanisms of thrombin. J. Thromb. Haemost..

[2] Wolberg A.S. (2007). Thrombin generation and fibrin clot structure. Blood Rev..

[3] Di Cera E. (2008). Thrombin. Mol. Asp. Med..

[4] Licari L.G., Kovacic J.P. (2009). Thrombin physiology and pathophysiology. J. Vet. Emerg. Crit. Care.

[5] Mazepa M., Hoffman M., Monroe D. (2013). Superactivated platelets: Thrombus regulators, thrombin generators, and potential clinical targets. Arterioscler. Thromb. Vasc. Biol..

[6] Davidson B.L. (2015). The association of direct thrombin inhibitor anticoagulants with cardiac thromboses. Chest.

[7] Di Cera E. (2007). Thrombin as procoagulant and anticoagulant. J. Thromb. Haemost..

[8] Bock P.E., Panizzi P., Verhamme I.M.A. (2007). Exosites in the substrate specificity of blood coagulation reactions. J. Thromb. Haemost..

[9] Crawley J.T.B., Zanardelli S., Chion C.K.N.K., Lane D.A. (2007). The central role of thrombin in hemostasis. J. Thromb. Haemost..

[10] Huntington J.A., Baglin T.P. (2003). Targeting thrombin—Rational drug design from natural mechanisms. Trends Pharmacol. Sci..

[11] Hirsh J. (2003). Current anticoagulant therapy—Unmet clinical needs. Thromb. Res..

[12] Gómez Outes A., Suárez Gea M.L., Pozo Hernández C., Lecumberri R., Rocha E., Vargas Castrillón E. (2011). New parenteral anticoagulants in development. Ther. Adv. Cardiovasc. Dis..

[13] Zavyalova E., Ustinov N., Golovin A., Pavlova G., Kopylov A. (2016). G-quadruplex aptamers to human thrombin versus other direct thrombin inhibitors: The focus on mechanism of action and drug efficiency as anticoagulants. Curr. Med. Chem..

[14] Roxo C., Kotkowiak W., Pasternak A. (2019). G-quadruplex forming aptamers-characteristics, applications, and perspectives. Molecules.

[15] Aviñó A., Fàbrega C., Tintoré M., Eritja R. (2012). Thrombin binding aptamer, more than a simple aptamer: Chemically modified derivatives and biomedical applications. Curr. Pharm. Des..

[16] Musumeci D., Montesarchio D. (2012). Polyvalent nucleic acid aptamers and modulation of their activity: A focus on the thrombin binding aptamer. Pharmacol. Ther..

[17] Platella C., Riccardi C., Montesarchio D., Roviello G.N., Musumeci D. (2017). G-quadruplex-based aptamers against protein targets in therapy and diagnostics. Biochim. Biophys. Acta Gen. Subj..

[18] Bock L.C., Griffin L.C., Latham J.A., Vermaas E.H., Toole J.J. (1992). Selection of single-stranded DNA molecules that bind and inhibit human thrombin. Nature.

[19] Macaya R.F., Schultze P., Smith F.W., Roe J.A., Feigon J. (1993). Thrombin-binding DNA aptamer forms a unimolecular quadruplex structure in solution. Proc. Natl. Acad. Sci. USA.

[20] Padmanabhan K., Padmanabhan K.P., Ferrara J.D., Sadler J.E., Tulinsky A. (1993). The structure of α-thrombin inhibited by a 15-mer single-stranded DNA aptamer. J. Biol. Chem..

[21] Nimjee S.M., Rusconi C.P., Harrington R.A., Sullenger B.A. (2005). The potential of aptamers as anticoagulants. Trends Cardiovasc. Med..

[22] Russo Krauss I., Merlino A., Giancola C., Randazzo A., Mazzarella L., Sica F. (2011). Thrombin-aptamer recognition: A revealed ambiguity. Nucleic Acids Res..

[23] Russo Krauss I., Merlino A., Randazzo A., Novellino E., Mazzarella L., Sica F. (2012). High-resolution structures of two complexes between thrombin and thrombin-binding aptamer shed light on the role of cations in the aptamer inhibitory activity. Nucleic Acids Res..

[24] Schwienhorst A. (2006). Direct thrombin inhibitors—A survey of recent developments. Cell. Mol. Life Sci..

[25] Gatto B., Palumbo M., Sissi C. (2009). Nucleic acid aptamers based on the G-quadruplex structure: Therapeutic and diagnostic potential. Curr. Med. Chem..

[26] Nimjee S.M., White R.R., Becker R.C., Sullenger B.A. (2017). Aptamers as therapeutics. Annu. Rev. Pharmacol. Toxicol..

[27] Zhou J., Rossi J. (2017). Aptamers as targeted therapeutics: Current potential and challenges. Nat. Rev. Drug Discov..

[28] Deng B., Lin Y., Wang C., Li F., Wang Z., Zhang H., Li X.F., Le X.C. (2014). Aptamer binding assays for proteins: The thrombin example-a review. Anal. Chim. Acta.

[29] Musumeci D., Platella C., Riccardi C., Moccia F., Montesarchio D. (2017). Fluorescence sensing using DNA aptamers in cancer research and clinical diagnostics. Cancers.

[30] Ni X., Castanares M., Mukherjee A., Lupold S.E. (2011). Nucleic acid aptamers: Clinical applications and promising new horizons. Curr. Med. Chem..

[31] Nimjee S.M., Povsic T.J., Sullenger B.A., Becker R.C. (2016). Translation and clinical development of antithrombotic aptamers. Nucleic Acid Ther..

[32] Riccardi C., Meyer A., Vasseur J.J., Russo Krauss I., Paduano L., Oliva R., Petraccone L., Morvan F., Montesarchio D. (2019). Stability is not everything: The case of the cyclization of the thrombin binding aptamer. ChemBioChem.

[33] Riccardi C., Meyer A., Vasseur J.J., Russo Krauss I., Paduano L., Morvan F., Montesarchio D. (2020). Fine-tuning the properties of the thrombin binding aptamer through cyclization: Effect of the 5′-3′ connecting linker on the aptamer stability and anticoagulant activity. Bioorg. Chem..

[34] Michalowski D., Chitima-Matsiga R., Held D.M., Burke D.H. (2008). Novel bimodular DNA aptamers with guanosine quadruplexes inhibit phylogenetically diverse HIV-1 reverse transcriptases. Nucleic Acids Res..

[35] Li T., Wang E., Dong S. (2009). A grafting strategy for the design of improved G-quadruplex aptamers and high-activity DNAzymes. PLoS ONE.

[36] Mazurov A.V., Titaeva E.V., Khaspekova S.G., Storojilova A.N., Spiridonova V.A., Kopylov A.M., Dobrovolsky A.B. (2011). Characteristics of a new DNA aptamer, direct inhibitor of thrombin. Bull. Exp. Biol. Med..

[37] Spiridonova V.A., Barinova K.V., Glinkina K.A., Melnichuk A.V., Gainutdynov A.A., Safenkova I.V., Dzantiev B.B. (2015). A family of DNA aptamers with varied duplex region length that forms complexes with thrombin and prothrombin. FEBS Lett..

[38] Buff M.C.R., Schäfer F., Wulffen B., Müller J., Pötzsch B., Heckel A., Mayer G. (2009). Dependence of aptamer activity on opposed terminal extensions: Improvement of light-regulation efficiency. Nucleic Acids Res..

[39] Wagner-Whyte J., Khuri S.F., Preiss J.R., Kurz J.C., Olson K., Hatala P., Boomer R.M., Fraone J.M., Brosnan N., Makim A. (2007). Discovery of a potent, direct thrombin inhibiting aptamer. J. Thromb. Haemost..

[40] ARCA Biopharma Study of NU172 as Anticoagulation in Patients Undergoing Off-Pump CABG Surgery. https://clinicaltrials.gov/ct2/show/NCT00808964.

[41] Keefe A.D., Pai S., Ellington A. (2010). Aptamers as therapeutics. Nat. Rev. Drug Discov..

[42] Waters E., Richardson J., Schaub R., Kurz J. (2009). Effect of NU172 and bivalirudin on ecarin clotting time in human plasma and whole blood. J. Thromb. Haemost..

[43] Zavyalova E., Golovin A., Pavlova G., Kopylov A. (2013). Module-activity relationship of G-quadruplex based DNA aptamers for human thrombin. Curr. Med. Chem..

[44] Zavyalova E., Ustinov N., Kopylov A. (2020). Exploring the efficiency of thrombin inhibitors with a quantitative model of the coagulation cascade. FEBS Lett..

[45] Trapaidze A., Hérault J.P., Herbert J.M., Bancaud A., Gué A.M. (2016). Investigation of the selectivity of thrombin-binding aptamers for thrombin titration in murine plasma. Biosens. Bioelectron..

[46] Wakui K., Yoshitomi T., Yamaguchi A., Tsuchida M., Saito S., Shibukawa M., Furusho H., Yoshimoto K. (2019). Rapidly neutralizable and highly anticoagulant thrombin-binding DNA aptamer discovered by MACE SELEX. Mol. Ther. Nucleic Acids.

[47] Rangnekar A., Nash J.A., Goodfred B., Yingling Y.G., LaBean T.H. (2016). Design of potent and controllable anticoagulants using DNA aptamers and nanostructures. Molecules.

[48] Zavyalova E., Tagiltsev G., Reshetnikov R., Arutyunyan A., Kopylov A. (2016). Cation coordination alters the conformation of a thrombin-binding G-quadruplex DNA aptamer that affects inhibition of thrombin. Nucleic Acid Ther..

[49] Russo Krauss I., Napolitano V., Petraccone L., Troisi R., Spiridonova V., Mattia C.A., Sica F. (2018). Duplex/quadruplex oligonucleotides: Role of the duplex domain in the stabilization of a new generation of highly effective anti-thrombin aptamers. Int. J. Biol. Macromol..

[50] Zavyalova E.G., Legatova V.A., Alieva R.S., Zalevsky A.O., Tashlitsky V.N., Arutyunyan A.M., Kopylov A.M. (2019). Putative mechanisms underlying high inhibitory activities of bimodular DNA aptamers to thrombin. Biomolecules.

[51] De Fenza A.M., Eremeeva E., Troisi R., Esposito A., Sica F., Herdewijn P., D’Alonzo D., Guaragna A. (2020). Structure-activity relationship study of a potent α-thrombin binding aptamer incorporating hexitol nucleotides. Chemistry.

[52] Troisi R., Napolitano V., Spiridonova V., Russo Krauss I., Sica F. (2018). Several structural motifs cooperate in determining the highly effective anti-thrombin activity of NU172 aptamer. Nucleic Acids Res..

[53] Russo Krauss I., Spiridonova V., Pica A., Napolitano V., Sica F. (2016). Different duplex/quadruplex junctions determine the properties of anti-thrombin aptamers with mixed folding. Nucleic Acids Res..

[54] Pourceau G., Meyer A., Vasseur J.J., Morvan F. (2009). Azide solid support for 3′-conjugation of oligonucleotides and their circularization by click chemistry. J. Org. Chem..

[55] Edupuganti O.P., Defrancq E., Dumy P. (2003). Head-to-tail oxime cyclization of oligodeoxynucleotides for the efficient synthesis of circular DNA analogues. J. Org. Chem..

[56] Meyer A., Vasseur J.J., Dumy P., Morvan F. (2017). Phthalimide–oxy derivatives for 3′- or 5′-conjugation of oligonucleotides by oxime ligation and circularization of DNA by “bis- or tris-click” oxime ligation. Eur. J. Org. Chem..

[57] Gerland B., Goudot A., Pourceau G., Meyer A., Dugas V., Cecioni S., Vidal S., Souteyrand E., Vasseur J.J., Chevolot Y. (2012). Synthesis of a library of fucosylated glycoclusters and determination of their binding toward pseudomonas aeruginosa lectin B (PA-IIL) using a DNA-based carbohydrate microarray. Bioconj. Chem..

[58] Casoni F., Dupin L., Vergoten G., Meyer A., Ligeour C., Géhin T., Vidal O., Souteyrand E., Vasseur J.J., Chevolot Y. (2014). The influence of the aromatic aglycon of galactoclusters on the binding of LecA: A case study with O-phenyl, S-phenyl, O-benzyl, S-benzyl, O-biphenyl and O-naphthyl aglycons. Org. Biomol. Chem..

[59] Noël M., Clément-Blanc C., Meyer A., Vasseur J.J., Morvan F. (2019). Solid supports for the synthesis of 3′-aminooxy deoxy- or ribo-oligonucleotides and their 3′-conjugation by oxime ligation. J. Org. Chem..

[60] Largy E., Mergny J.L., Gabelica V. (2016). Role of alkali metal ions in G-quadruplex nucleic acid structure and stability. Metal Ions Life Sci..

[61] Largy E., Marchand A., Amrane S., Gabelica V., Mergny J.L. (2016). Quadruplex turncoats: Cation-dependent folding and stability of quadruplex-DNA double switches. J. Am. Chem. Soc..

[62] Bhattacharyya D., Arachchilage G.M., Basu S. (2016). Metal cations in G-quadruplex folding and stability. Front. Chem..

[63] Mergny J.-L., Li J., Lacroix L., Amrane S., Chaires J.B. (2005). Thermal difference spectra: A specific signature for nucleic acid structures. Nucleic Acids Res..

[64] Mergny J.-L., Lacroix L. (2009). UV Melting of G-quadruplexes. Curr. Protoc. Nucleic Acid Chem..

[65] Karsisiotis A.I., Hessari N.M.A., Novellino E., Spada G.P., Randazzo A., Webba da Silva M. (2011). Topological characterization of nucleic acid G-quadruplexes by UV absorption and circular dichroism. Angew. Chem. Int. Ed. Eng..

[66] Malgowska M., Gudanis D., Teubert A., Dominiak G., Gdaniec Z. (2012). How to study G-quadruplex structures. J. Biotechnol. Comput. Biol. Bionanotechnol..

[67] Kotkowiak W., Wengel J., Scotton C.J., Pasternak A. (2019). Improved RE31 analogues containing modified nucleic acid monomers: Thermodynamic, structural, and biological effects. J. Med. Chem..

[68] Dolinnaya N.G., Yuminova A.V., Spiridonova V.A., Arutyunyan A.M., Kopylov A.M. (2012). Coexistence of G-quadruplex and duplex domains within the secondary structure of 31-mer DNA thrombin-binding aptamer. J. Biomol. Struct. Dyn..

[69] Mergny J.-L., Phan A., Lacroix L. (1998). Following G-quartet formation by UV-spectroscopy. FEBS Lett..

[70] Rachwal P.A., Fox K.R. (2007). Quadruplex melting. Methods.

[71] Tan Z.J., Chen S.J. (2006). Nucleic acid helix stability: Effects of salt concentration, cation valence and size, and chain length. Biophys. J..

[72] Dapić V., Abdomerović V., Marrington R., Peberdy J., Rodger A., Trent J.O., Bates P.J. (2003). Biophysical and biological properties of quadruplex oligodeoxyribonucleotides. Nucleic Acids Res..

[73] Paramasivan S., Rujan I., Bolton P.H. (2007). Circular dichroism of quadruplex DNAs: Applications to structure, cation effects and ligand binding. Methods.

[74] Kypr J., Kejnovská I., Renčiuk D., Vorlíčková M. (2009). Circular dichroism and conformational polymorphism of DNA. Nucleic Acids Res..

[75] Masiero S., Trotta R., Pieraccini S., De Tito S., Perone R., Randazzo A., Spada G.P. (2010). A non-empirical chromophoric interpretation of CD spectra of DNA G-quadruplex structures. Org. Biomol. Chem..

[76] Vorlíčková M., Kejnovská I., Sagi J., Renčiuk D., Bednářová K., Motlová J., Kypr J. (2012). Circular dichroism and guanine quadruplexes. Methods.

[77] Randazzo A., Spada G.P., Webba da Silva M. (2013). Circular dichroism of quadruplex structures. Top. Curr. Chem..

[78] Del Villar-Guerra R., Gray R.D., Chaires J.B. (2017). Characterization of quadruplex DNA structure by circular dichroism. Curr. Protoc. Nucleic Acid Chem..

[79] Del Villar-Guerra R., Trent J.O., Chaires J.B. (2018). G-quadruplex secondary structure obtained from circular dichroism spectroscopy. Angew. Chem. Int. Ed. Eng..

[80] Hardin C.C., Watson T., Corregan M., Bailey C. (1992). Cation-dependent transition between the quadruplex and Watson-Crick hairpin forms of d(CGCG_3_GCG). Biochemistry.

[81] Hud N.V., Smith F.W., Anet F.A.L., Feigon J. (1996). The selectivity for K^+^ versus Na^+^ in DNA quadruplexes is dominated by relative free energies of hydration: A thermodynamic analysis by ^1^H NMR. Biochemistry.

[82] Poniková S., Antalík M., Hianik T. (2008). A circular dichroism study of the stability of guanine quadruplexes of thrombin DNA aptamers at presence of K^+^ and Na^+^ ions. Gen. Physiol. Biophys..

[83] Saintomé C., Amrane S., Mergny J.L., Alberti P. (2016). The exception that confirms the rule: A higher-order telomeric G-quadruplex structure more stable in sodium than in potassium. Nucleic Acids Res..

[84] Zaitseva M., Kaluzhny D., Shchyolkina A., Borisova O., Smirnov I., Pozmogova G. (2010). Conformation and thermostability of oligonucleotide d(GGTTGGTGTGGTTGG) containing thiophosphoryl internucleotide bonds at different positions. Biophys. Chem..

[85] Marky L.A., Breslauer K.J. (1987). Calculating thermodynamic data for transitions of any molecularity from equilibrium melting curves. Biopolymers.

[86] Mergny J.L., Lacroix L. (2003). Analysis of thermal melting curves. Oligonucleotides.

[87] Petersheim M., Turner D.H. (1983). Base-stacking and base-pairing contributions to helix stability: Thermodynamics of double-helix formation with CCGG, CCGGp, CCGGAp, ACCGGp, CCGGUp, and ACCGGUp. Biochemistry.

[88] Petraccone L., Erra E., Nasti L., Galeone A., Randazzo A., Mayol L., Barone G., Giancola C. (2003). Effect of a modified thymine on the structure and stability of [d(TGGGT)]_4_ quadruplex. Int. J. Biol. Macromol..

[89] Petraccone L., Erra E., Esposito V., Randazzo A., Mayol L., Nasti L., Barone G., Giancola C. (2004). Stability and structure of telomeric DNA sequences forming quadruplexes containing four G-tetrads with different topological arrangements. Biochemistry.

[90] Olsen C.M., Gmeiner W.H., Marky L.A. (2006). Unfolding of G-quadruplexes: Energetic, and ion and water contributions of G-quartet stacking. J. Phys. Chem. B.

[91] Petraccone L., Spink C., Trent J.O., Garbett N.C., Mekmaysy C.S., Giancola C., Chaires J.B. (2011). Structure and stability of higher-order human telomeric quadruplexes. J. Am. Chem. Soc..

[92] Riccardi C., Musumeci D., Russo Krauss I., Piccolo M., Irace C., Paduano L., Montesarchio D. (2018). Exploring the conformational behaviour and aggregation properties of lipid-conjugated AS1411 aptamers. Int. J. Biol. Macromol..

[93] Riccardi C., Russo Krauss I., Musumeci D., Morvan F., Meyer A., Vasseur J.J., Paduano L., Montesarchio D. (2017). Fluorescent thrombin binding aptamer-tagged nanoparticles for an efficient and reversible control of thrombin activity. ACS Appl. Mater. Interfaces.

[94] Kibbe W.A. (2007). OligoCalc: An online oligonucleotide properties calculator. Nucleic Acids Res..

[95] Petraccone L., Pagano B., Giancola C. (2012). Studying the effect of crowding and dehydration on DNA G-quadruplexes. Methods.

[96] Riccardi C., Musumeci D., Platella C., Gaglione R., Arciello A., Montesarchio D. (2020). Tuning the polymorphism of the anti-VEGF G-rich V7t1 aptamer by covalent dimeric constructs. Int. J. Mol. Sci..

[97] Moccia F., Riccardi C., Musumeci D., Leone S., Oliva R., Petraccone L., Montesarchio D. (2019). Insights into the G-rich VEGF-binding aptamer V7t1: When two G-quadruplexes are better than one!. Nucleic Acids Res..

[98] Martino L., Virno A., Randazzo A., Virgilio A., Esposito V., Giancola C., Bucci M., Cirino G., Mayol L. (2006). A new modified thrombin binding aptamer containing a 5′-5′ inversion of polarity site. Nucleic Acids Res..

[99] Coppola T., Varra M., Oliviero G., Galeone A., D’Isa G., Mayol L., Morelli E., Bucci M.R., Vellecco V., Cirino G. (2008). Synthesis, structural studies and biological properties of new TBA analogues containing an acyclic nucleotide. Bioorg. Med. Chem..

[100] Bonifacio L., Church F.C., Jarstfer M.B. (2008). Effect of locked-nucleic acid on a biologically active G-quadruplex. A structure-activity relationship of the thrombin aptamer. Int. J. Mol. Sci..

[101] Ying G.Q., Lu X.R., Mei J.F., Zhang Y., Chen J.S., Wang X.D., Ou Z.M., Yi Y. (2019). A structure-activity relationship of a thrombin-binding aptamer containing LNA in novel sites. Bioorg. Med. Chem..

[102] Baldrich E., O’Sullivan C.K. (2005). Ability of thrombin to act as molecular chaperone, inducing formation of quadruplex structure of thrombin-binding aptamer. Anal. Biochem..

