# Supplementary material for: Design, Synthesis and Characterization of Cyclic NU172 Analogues: A Biophysical and Biological Insight
Source: Int J Mol Sci. 2020 May 29;21(11):3860. doi: 10.3390/ijms21113860 (PMC7312020; doi:10.3390/ijms21113860)
Supplement: Supplementary file 1 [file ijms-21-03860-s001.pdf]

Supporting Information

Design, Synthesis and Characterization of Cyclic NU172 Analogues: A Biophysical and Biological Insight

Claudia Riccardi [1], Albert Meyer [2], Jean-Jacques Vasseur [2], Domenico Cavasso [1], Irene Russo Krauss [1,3], Luigi Paduano [1,3], François Morvan [2,*] and Daniela Montesarchio [1,*]

1 Department of Chemical Sciences, University of Naples Federico II, Via Cintia 21, I-80126 Naples, Italy; claudia.riccardi@unina.it (C.R.); domenicocavasso@gmail.com (D.C.); irene.russokrauss@unina.it (I.R.K.); luigi.paduano@unina.it (L.P.)

2 Institut des Biomolécules Max Mousseron, Univ. Montpellier, CNRS, ENSCM, 34095 Montpellier, France; albert.meyer@umontpellier.fr (A.M.); jean-jacques.vasseur@umontpellier.fr (J.-J.V.)

3 CSGI—Consorzio Interuniversitario per lo Sviluppo dei Sistemi a Grande Interfase, Via della Lastruccia 3, I-50019 Sesto Fiorentino, Italy

* Correspondence: francois.morvan@umontpellier.fr (F.M.); daniela.montesarchio@unina.it (D.M.)

Table of contents

Scheme S1. Synthesis of **cycNU172-EG2** and **cycNU172-EG3** **pag. S3**

Figure S1. HPLC profiles of crude oligonucleotides **6** and **7** **pag. S4**

Figure S2. HPLC and MALDI-TOF profiles of pure **cycNU172-EG2** **pag. S5**

Figure S3. HPLC and MALDI-TOF profiles of pure **cycNU172-EG3** **pag. S6**

Scheme S2. Synthesis of of **cycNU172-Ph** and **cycNU172-Pro** **pag. S7**

Figure S4. HPLC profile of crude oligonucleotide **11** **pag. S8**

Figure S5. HPLC and MALDI-TOF profiles of pure **cycNU172-Ph** **pag. S9**

Figure S6. HPLC and MALDI-TOF profiles of pure **cycNU172-Pro** **pag. S10**

Figure S7. 20 % denaturing polyacrylamide gel electrophoresis analysis **pag. S11**

Table S1. TDS factor analysis for the 15-mer NU **pag. S12**

Figure S8. Overlapped UV-melting profiles at 260 nm **pag. S13**

Figure S9. UV analysis of the 15-mer NU at 295 nm **pag. S14**

Figure S10. UV analysis of NU172 both at 295 and 260 nm **pag. S15**

Figure S11. UV analysis of **cycNU172-EG2** both at 295 and 260 nm **pag. S16**

Figure S12. UV analysis of **cycNU172-EG3** both at 295 and 260 nm **pag. S17**

Figure S13. UV analysis of **cycNU172-Ph** both at 295 and 260 nm **pag. S18**

Figure S14. UV analysis of **cycNU172-Pro** both at 295 and 260 nm **pag. S19**

Table S2. T_m values obtained by UV-monitored thermal experiments **pag. S20**

Table S3. Specific λ values observed in the CD spectra **pag. S21**

Table S4. Prediction of G4 topologies adopted by NU by SVD analysis **pag. S22**

Figure S15. CD difference spectrum of NU172 and NU in the K^+-rich buffer **pag. S23**

Figure S16. CD analysis of the 15-mer NU **pag. S24**

Figure S17. CD analysis of NU172 **pag. S25**

Figure S18. CD analysis of **cycNU172-EG2** **pag. S26**

Figure S19. CD analysis of **cycNU172-EG3** **pag. S27**

Figure S20. CD analysis of **cycNU172-Ph** **pag. S28**

Figure S21. CD analysis of **cycNU172-Pro** **pag. S29**

Table S5. T_m values obtained by CD-monitored thermal experiments **pag. S30**

Table S6. Standard thermodynamic parameters from van’t Hoff analysis **pag. S31**

Figure S22. Nuclease resistance experiments on NU172 and its cyclic derivatives **pag. S32**

References. **pag. S33**

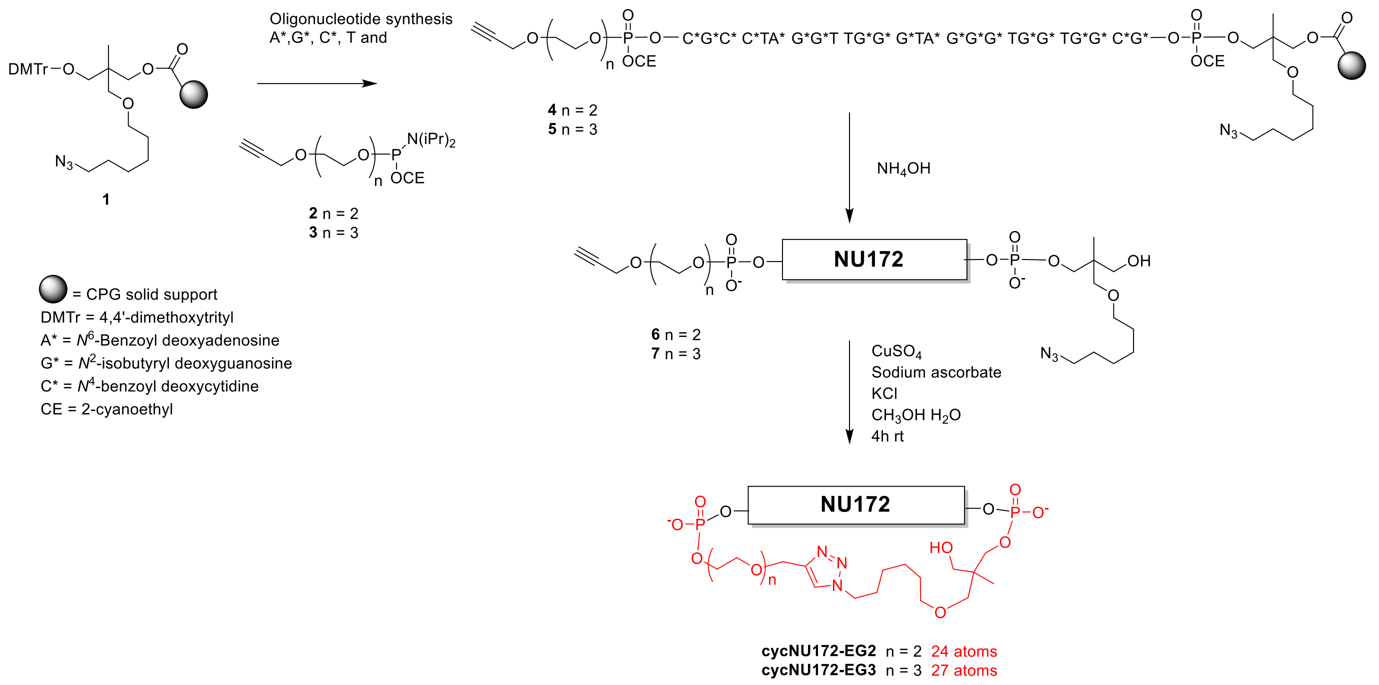

Scheme S1. Synthetic scheme for the preparation of **cycNU172-EG2** and **cycNU172-EG3** by CuAAC cyclization. NU172 sequence = 5'-CGCCTAGGTTGGGTAGGGTGGTGGCG-3'.

a)

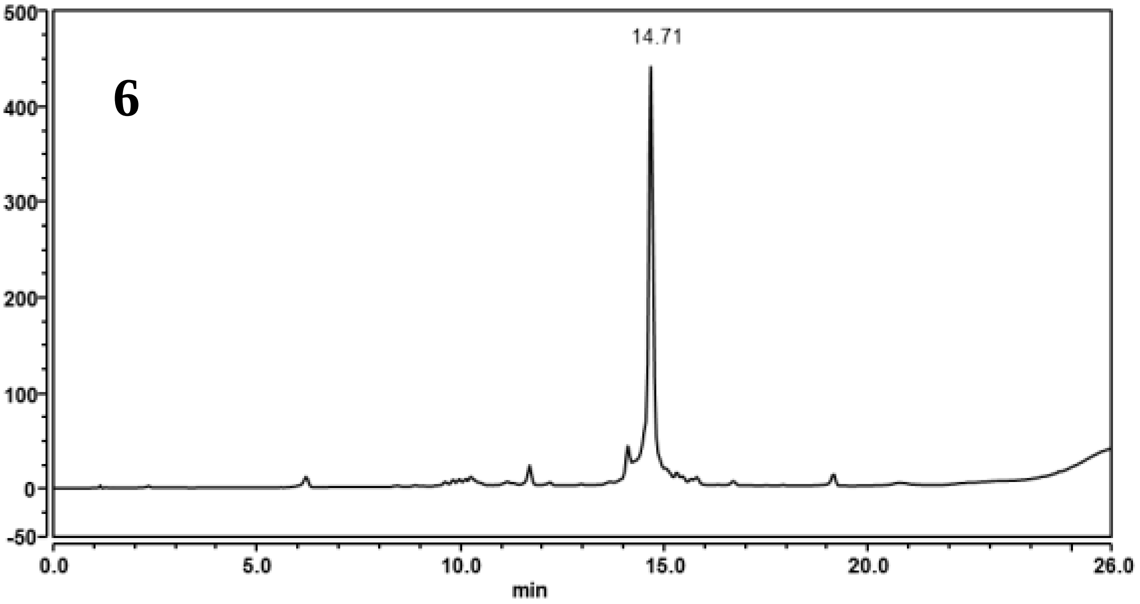

b)

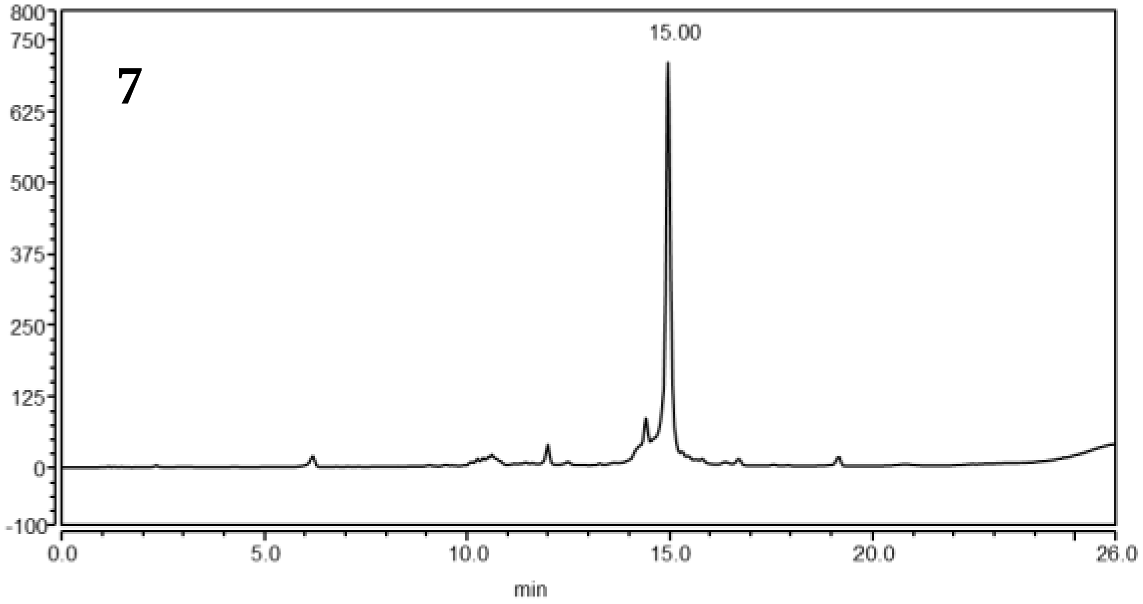

Figure S1. HPLC profiles of crude oligonucleotides **6** (**a**) and **7** (**b**), precursors respectively of **cycNU172-EG2** and **cycNU172-EG3**.

a)

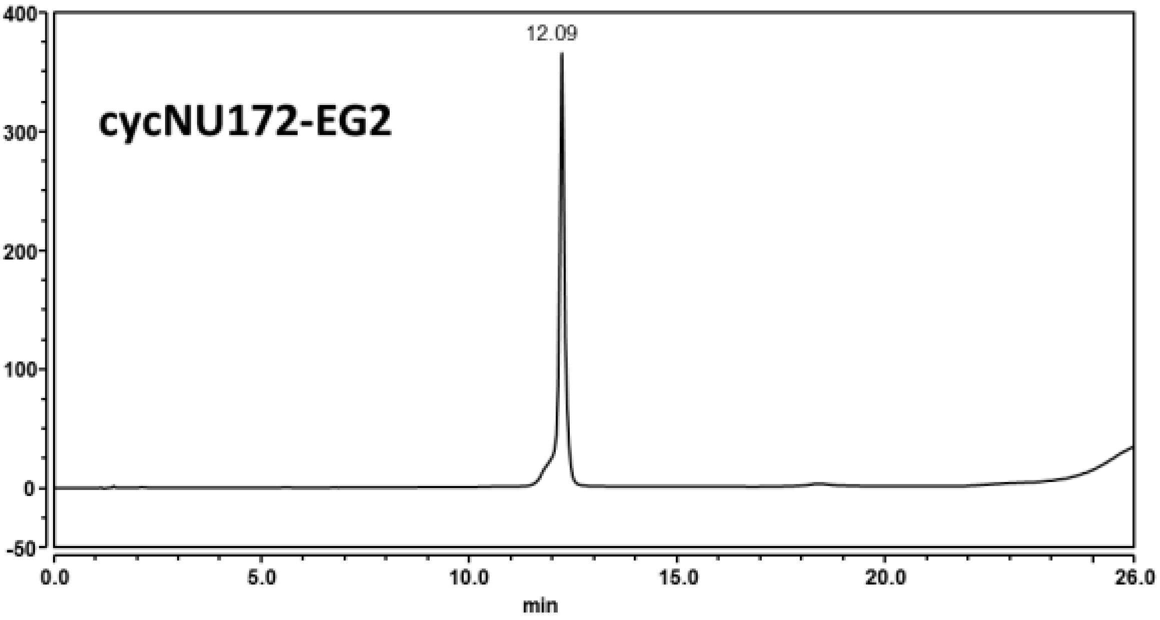

b)

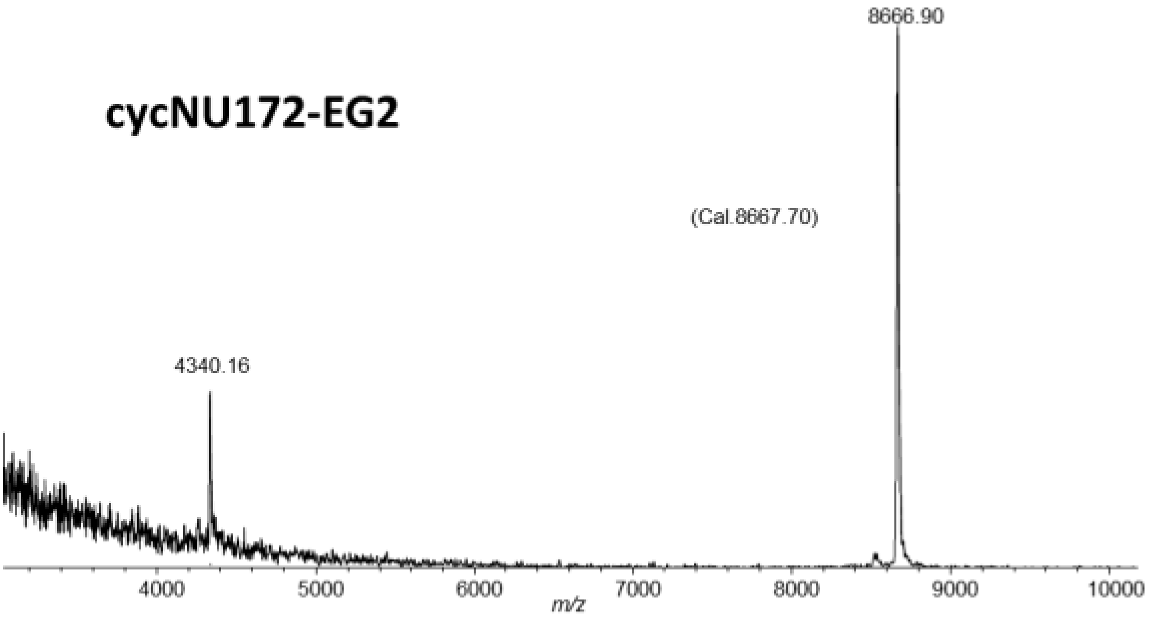

Figure S2. HPLC (**a**) and MALDI-TOF (**b**) profiles of pure **cycNU172-EG2**.

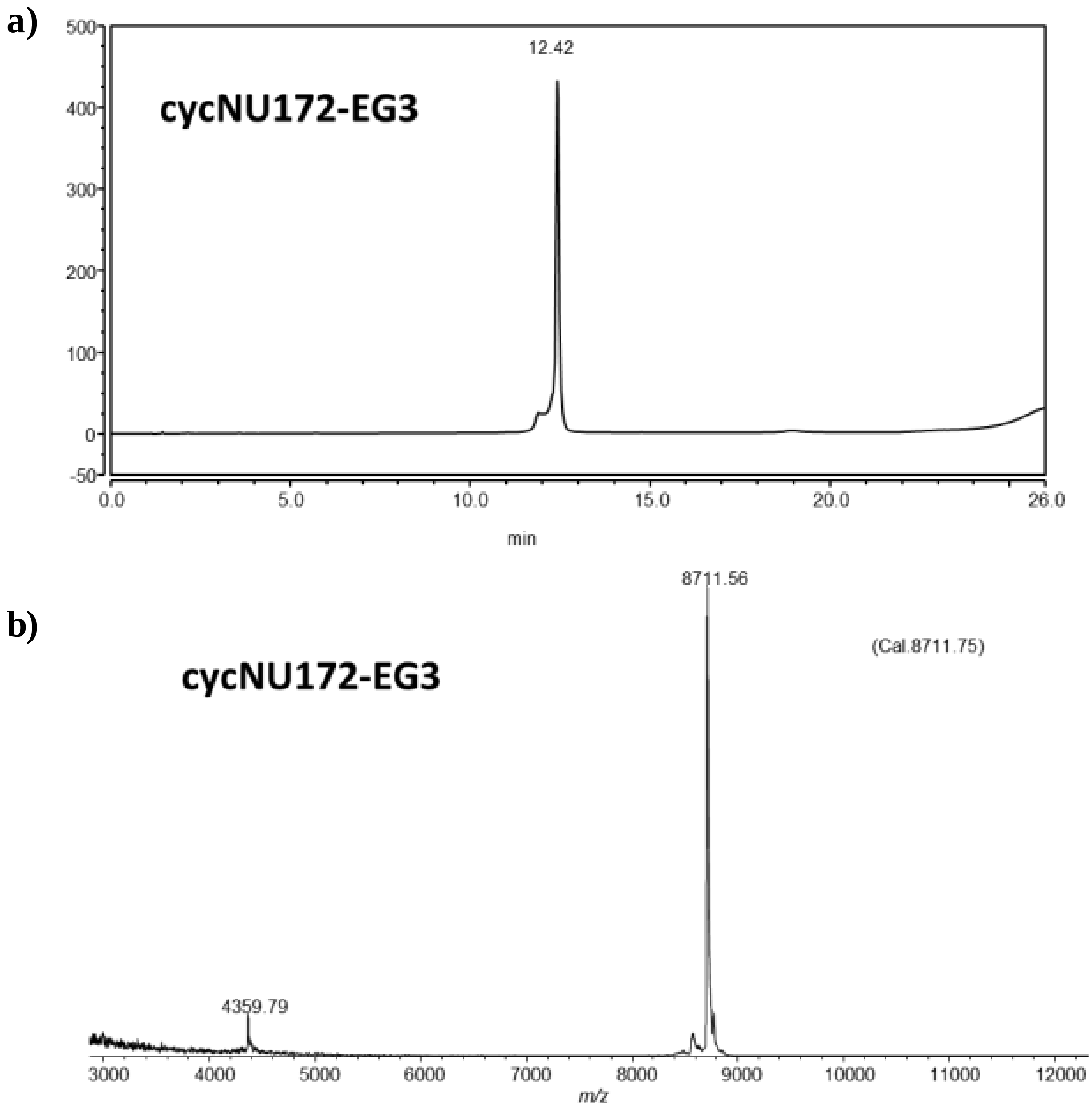

Figure S3. HPLC (**a**) and MALDI-TOF (**b**) profiles of pure **cycNU172-EG3**.

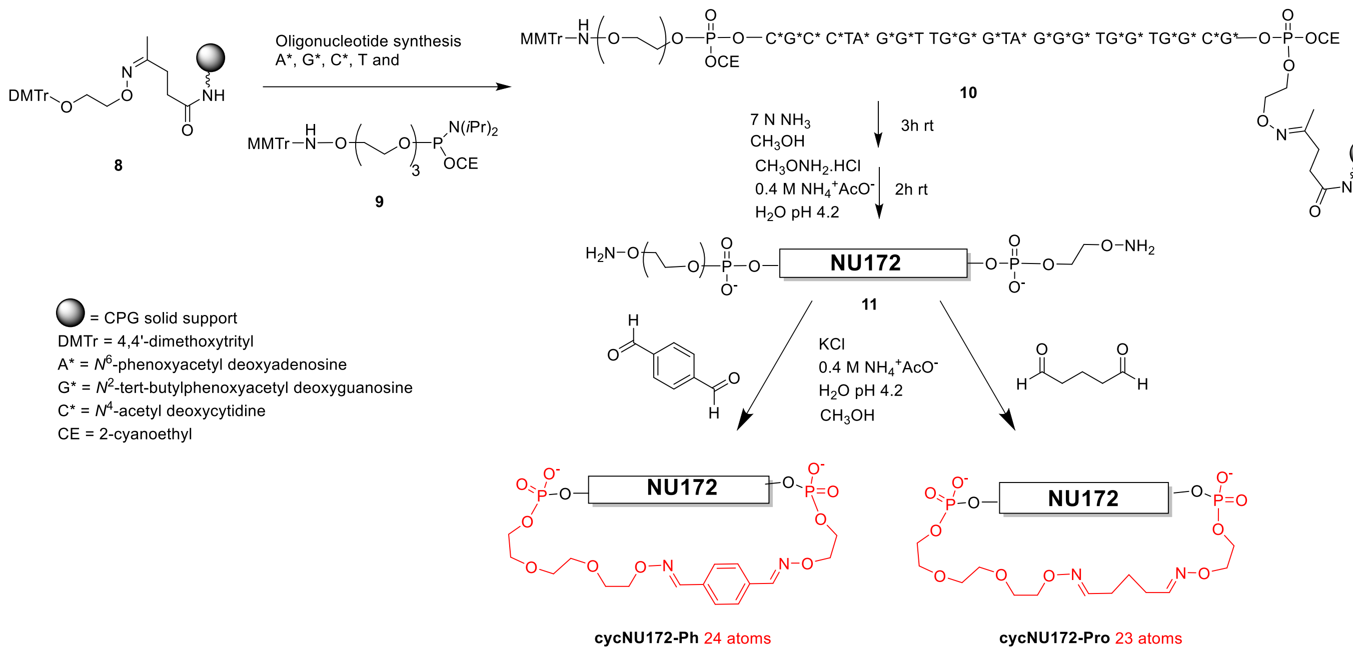

Scheme S2. Synthetic scheme for the preparation of **cycNU172-Ph** and **cycNU172-Pro**, obtained by bis-oxime cyclization. NU172 sequence = 5'-CGCCTAGGTTGGGTAGGGTGGTGGCG-3'.

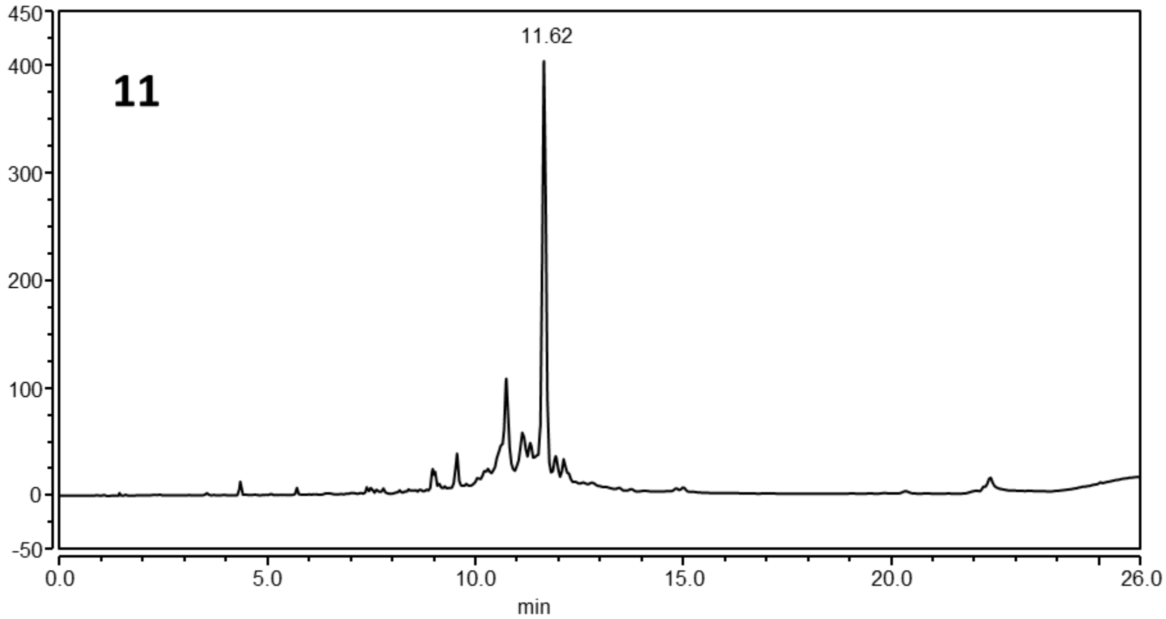

Figure S4. HPLC profile of crude oligonucleotide **11**, precursor of both **cycNU172-Ph** and **cycNU172-Pro**.

a)

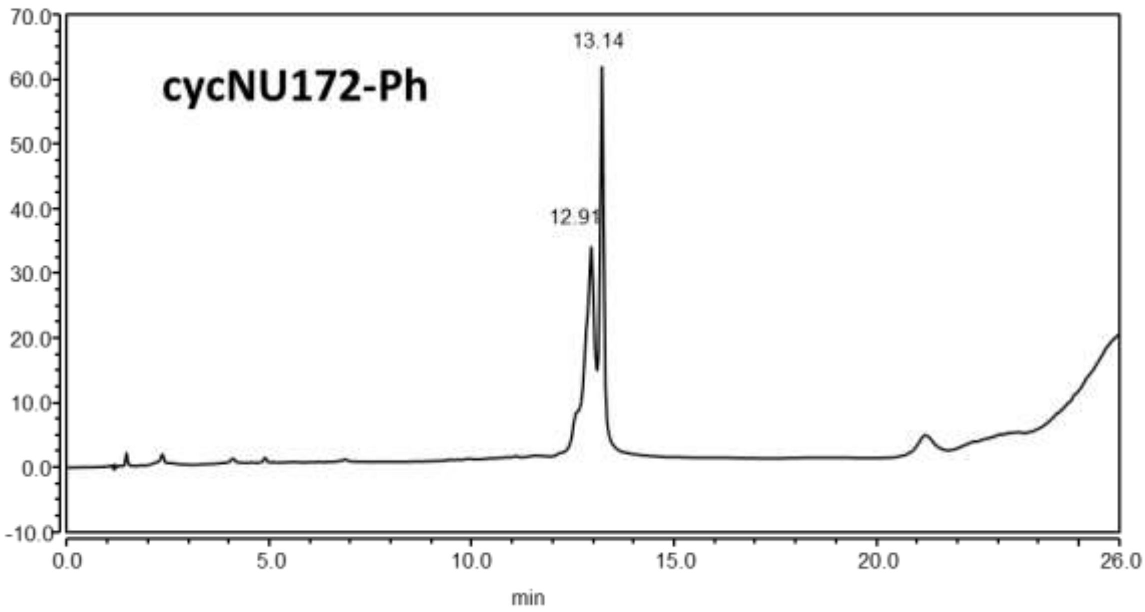

b)

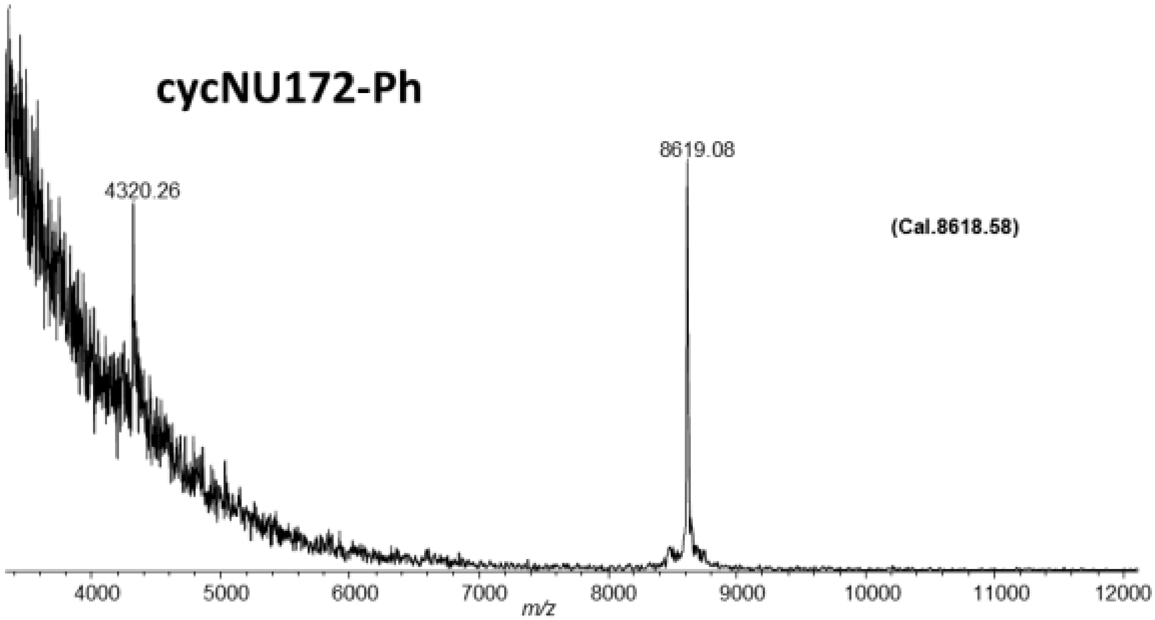

Figure S5. HPLC (**a**) and MALDI-TOF (**b**) profiles of pure **cycNU172-Ph**.

a)

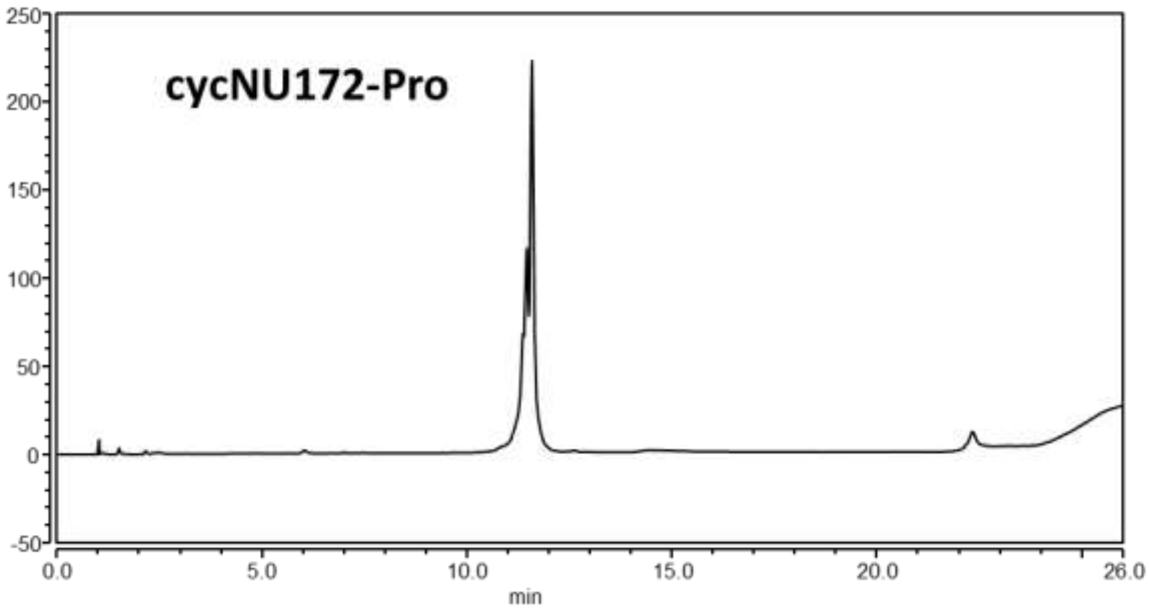

b)

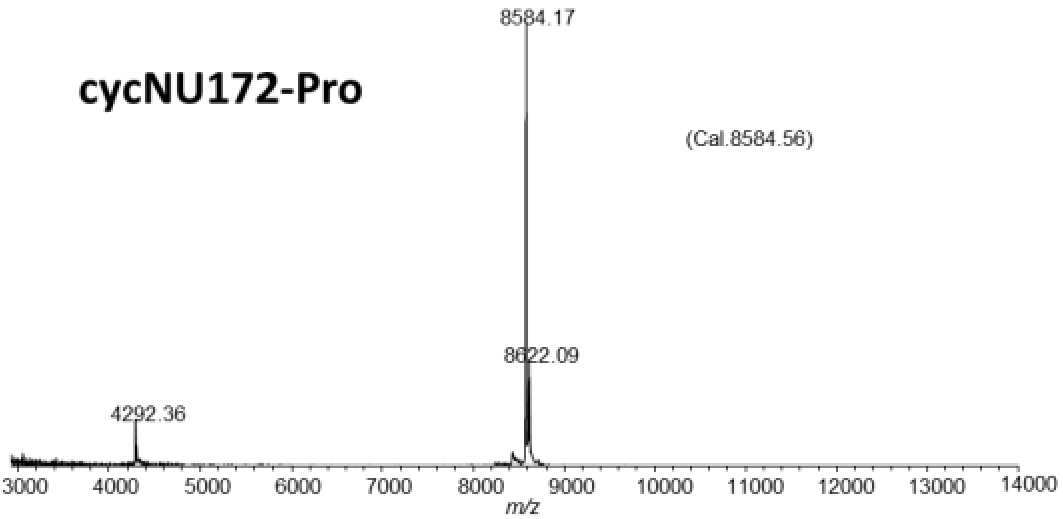

Figure S6. HPLC (**a**) and MALDI-TOF (**b**) profiles of pure **cycNU172-Pro**.

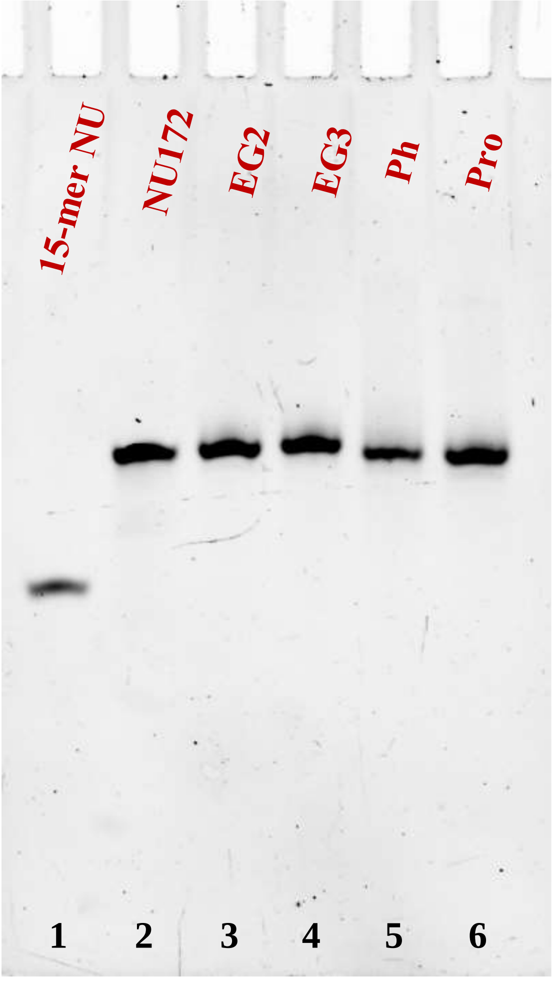

Figure S7. Representative 20 % denaturing polyacrylamide gel electrophoresis (8 M urea) at 4.5 μM sample concentration, run at constant 200 V at r.t. for 2.5 h in TBE 1X as running buffer. Lane 1: 15-mer NU; lane 2: NU172; lane 3: **cycNU172-EG2**; lane 4: **cycNU172-EG3**; lane 5: **cycNU172-Ph**; lane 6: **cycNU172-Pro**.

15-mer NU	TDS factors	
K^+-rich buffer		
$\Delta A_{240}/\Delta A_{295}$	$\Delta A_{255}/\Delta A_{295}$	$\Delta A_{275}/\Delta A_{295}$
0.5	0.1	0.5
Na^+-rich buffer		
$\Delta A_{240}/\Delta A_{295}$	$\Delta A_{255}/\Delta A_{295}$	$\Delta A_{275}/\Delta A_{295}$
1.2	0.6	1.3

Table S1. Ratios between values at different absorbance wavelengths as calculated from normalized TDS spectra for the 15-mer NU in both the selected phosphate buffer solutions, according to literature protocols.(1)

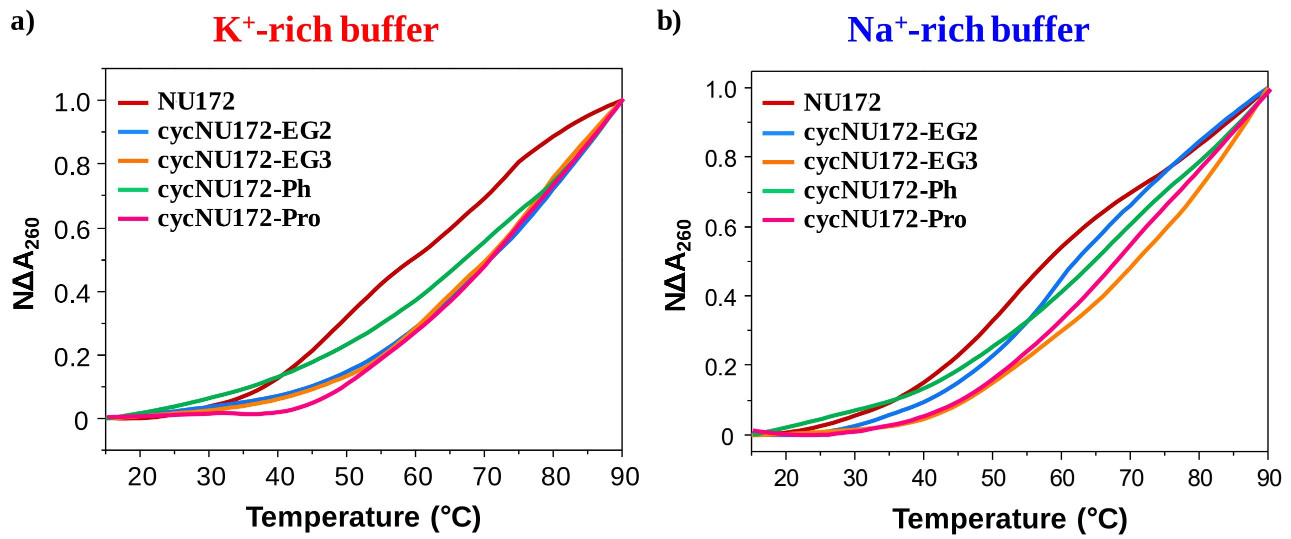

Figure S8. Representative UV-melting profiles of the cyclic NU172 derivatives (light blue, orange, green, and magenta lines, respectively for **cycNU172-EG2**, **cycNU172-EG3**, **cycNU172-Ph** and **cycNU172-Pro**), at 2 μM concentration in both the selected K^{+}- (**a**) and Na^{+}-rich (**b**) buffer solutions, in comparison with unmodified NU172 (dark red line). UV-melting profiles - recorded at 260 nm using a scan rate of 1 °C/min - are reported in terms of normalized absorbance as a function of the temperature.

15-mer NU

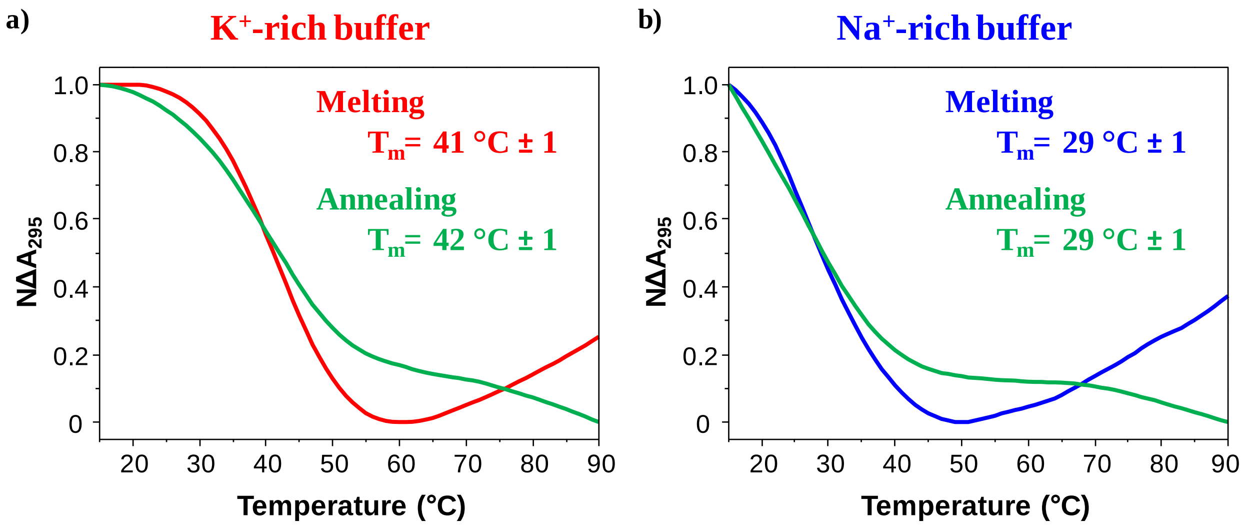

Figure S9. Representative normalized UV-melting and UV-annealing profiles of the 15-mer NU at 2 μM concentration in both the selected K^+- (**a**) and Na^+-rich (**b**) buffer solutions. The UV-monitored thermal curves were recorded following the signal at 295 nm in both saline conditions, with a temperature scan rate of 1 °C/min.

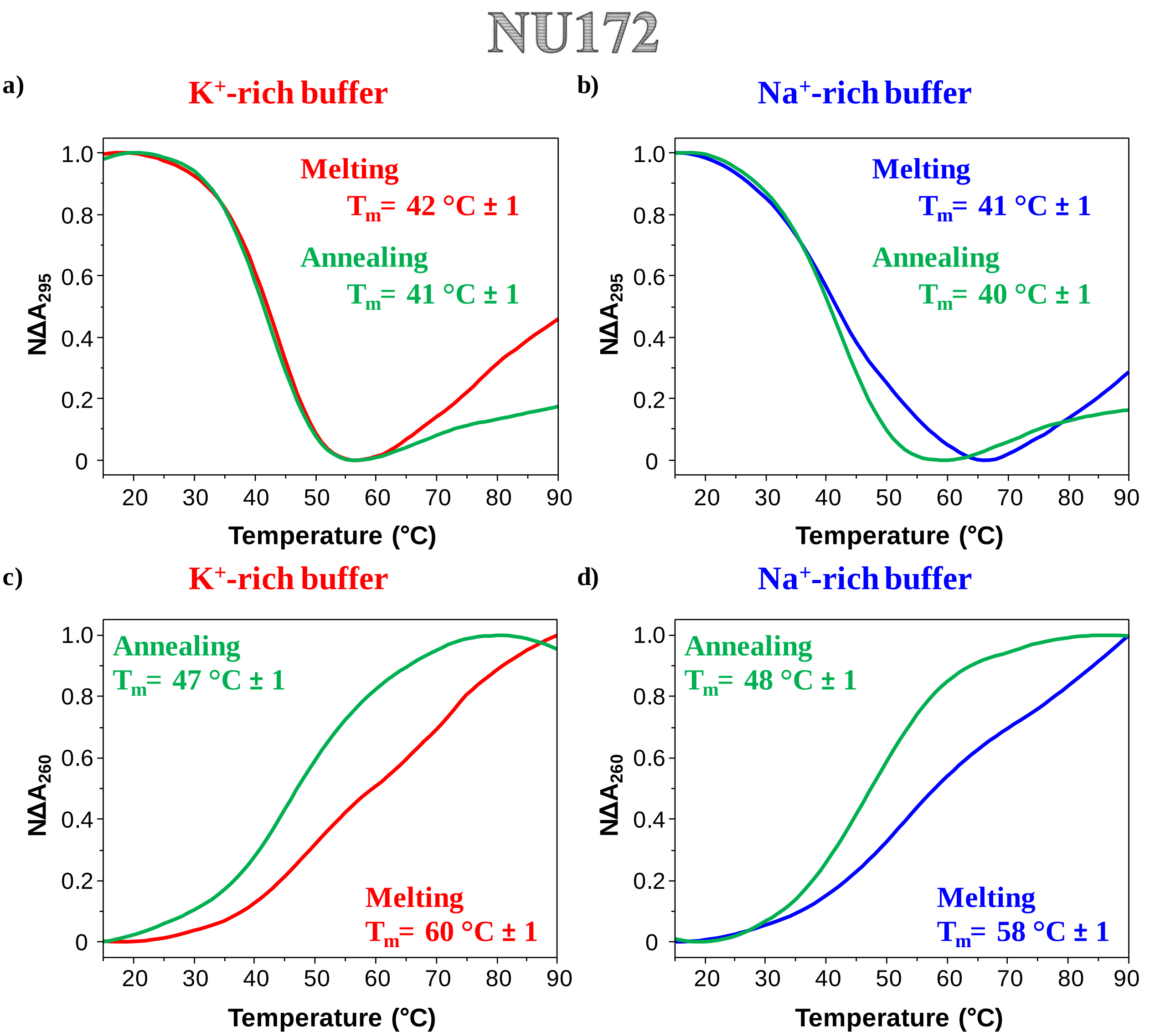

Figure S10. Representative normalized UV-melting and UV-annealing profiles of NU172 at 2 μM concentration in both the selected K^+- (**a**, **c**) and Na^+-rich (**b**, **d**) buffer solutions. The UV-monitored thermal curves were recorded following the signal at 295 (**a**, **b**) or 260 (**c**, **d**) nm in both saline conditions, with a temperature scan rate of 1 °C/min.

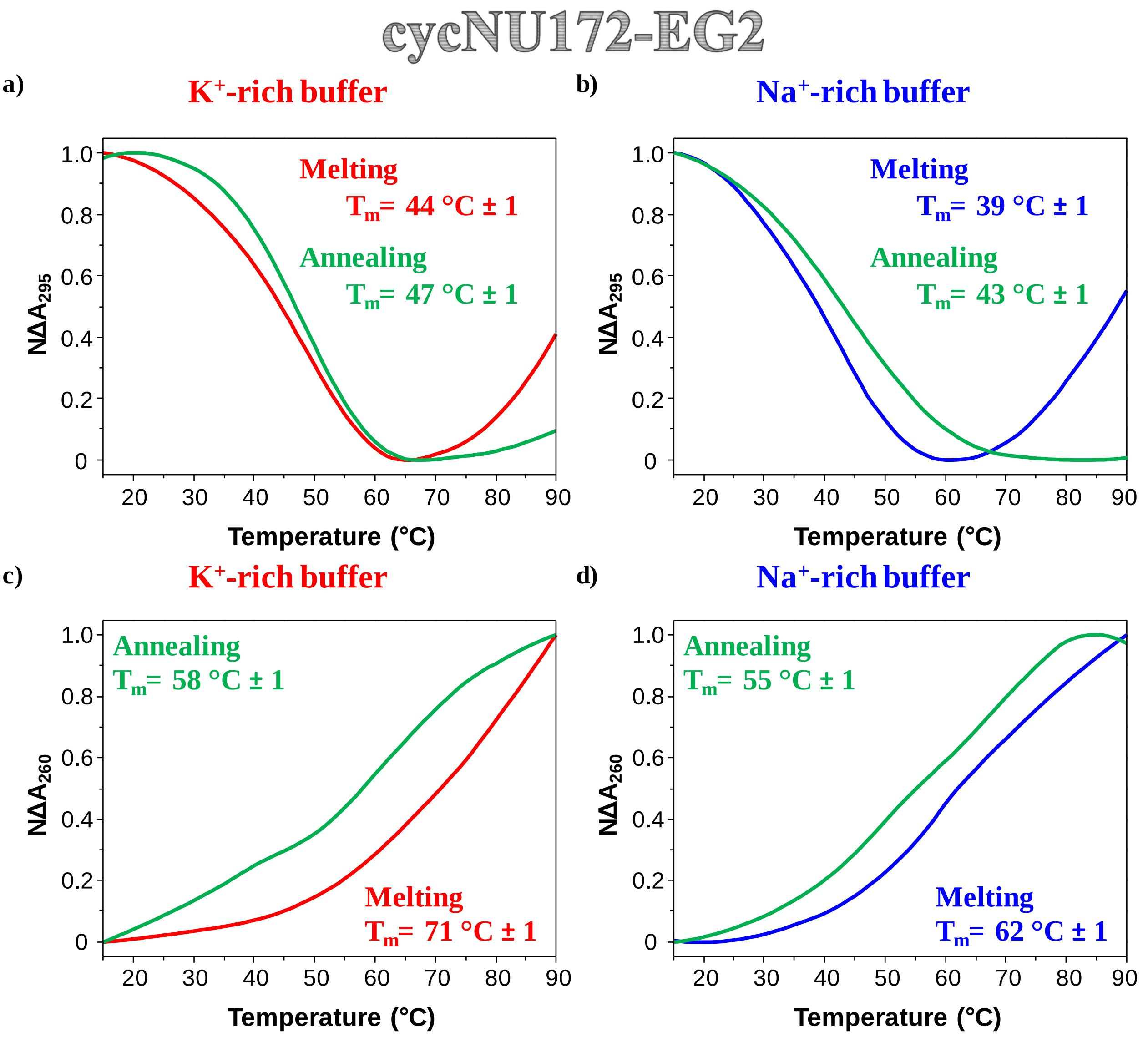

Figure S11. Representative normalized UV-melting and UV-annealing profiles of **cycNU172-EG2** at 2 μM concentration in both the selected K^+- (**a**, **c**) and Na^+-rich (**b**, **d**) buffer solutions. The UV-monitored thermal curves were recorded following the signal at 295 (**a**, **b**) or 260 (**c**, **d**) nm in both saline conditions, with a temperature scan rate of 1 °C/min.

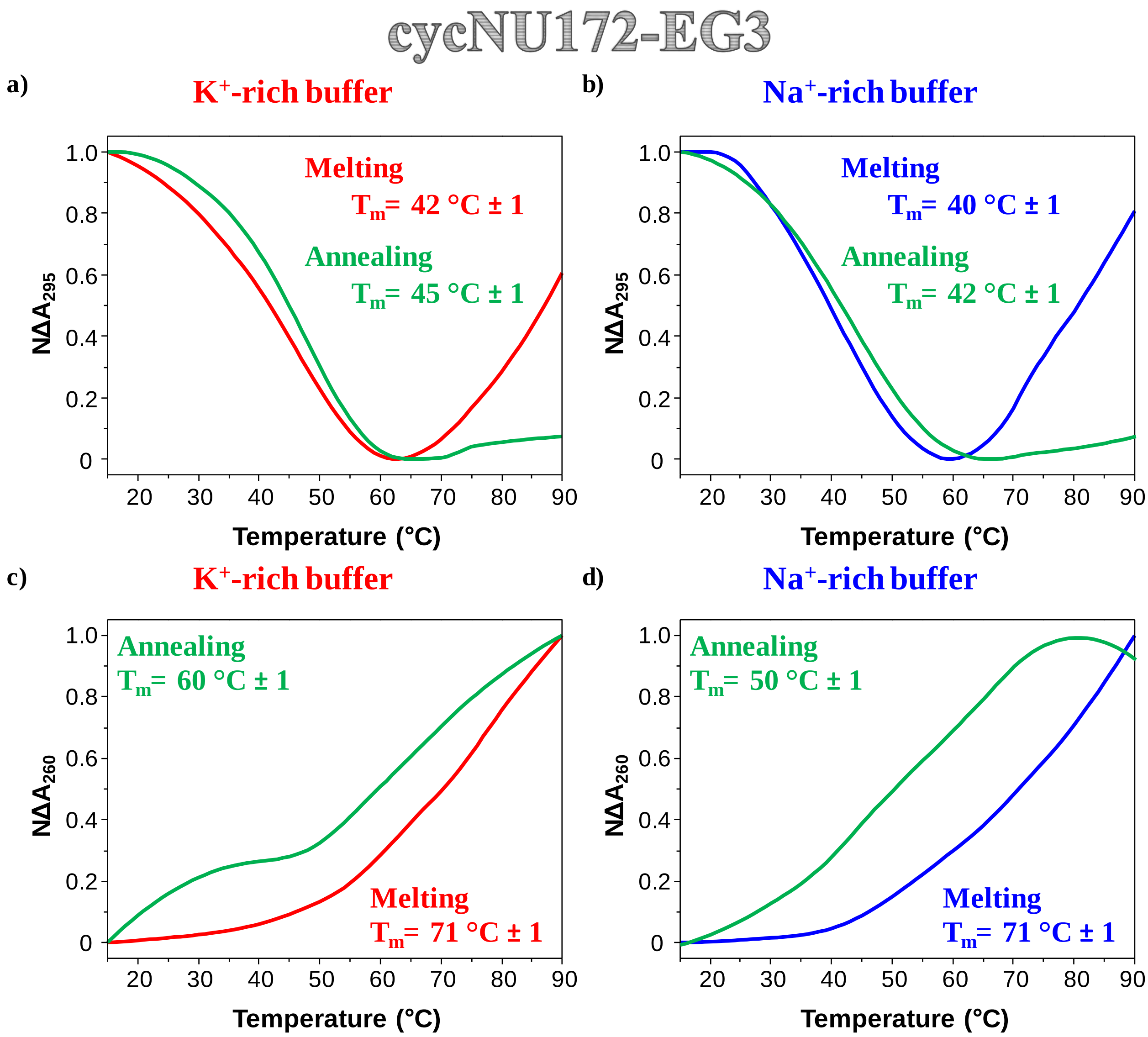

Figure S12. Representative normalized UV-melting and UV-annealing profiles of **cycNU172-EG3** at 2 μM concentration in both the selected K^+- (**a**, **c**) and Na^+-rich (**b**, **d**) buffer solutions. The UV-monitored thermal curves were recorded following the signal at 295 (**a**, **b**) or 260 (**c**, **d**) nm in both saline conditions, with a temperature scan rate of 1 °C/min.

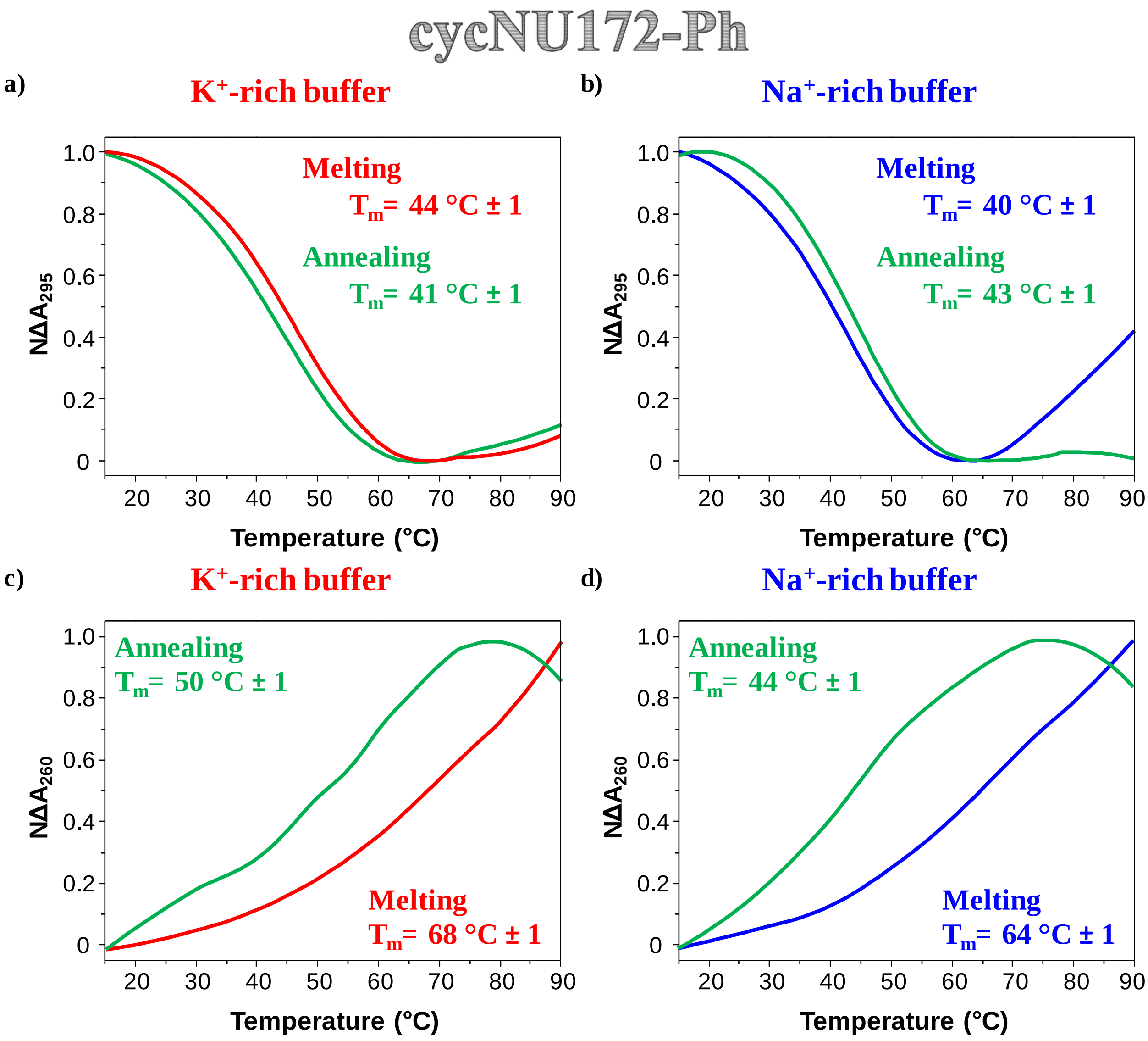

Figure S13. Representative normalized UV-melting and UV-annealing profiles of **cycNU172-Ph** at 2 μM concentration in both the selected K^+- (**a**, **c**) and Na^+-rich (**b**, **d**) buffer solutions. The UV-monitored thermal curves were recorded following the signal at 295 (**a**, **b**) or 260 (**c**, **d**) nm in both saline conditions, with a temperature scan rate of 1 °C/min.

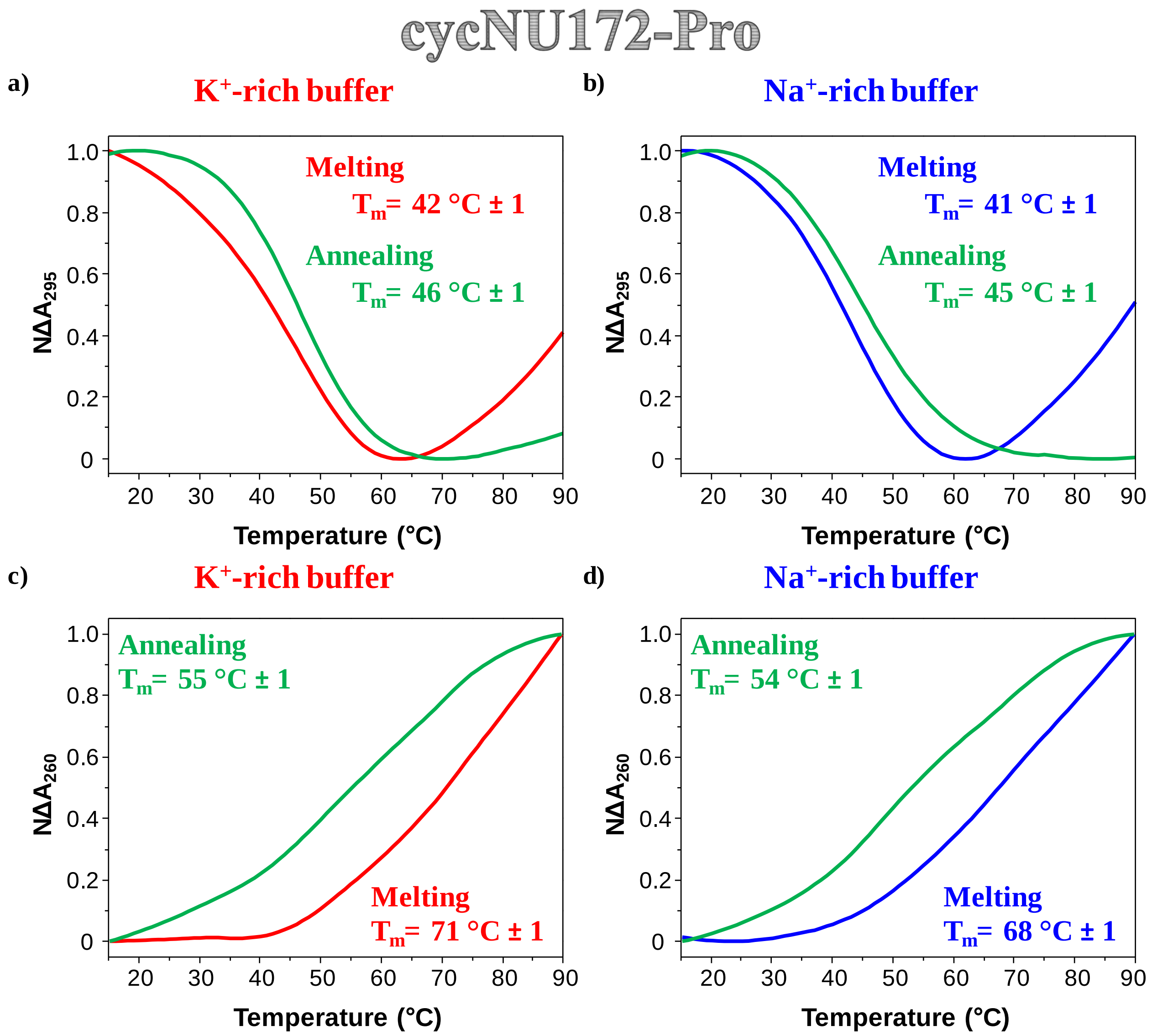

Figure S14. Representative normalized UV-melting and UV-annealing profiles of **cycNU172-Pro** at 2 μM concentration in both the selected K^+- (**a**, **c**) and Na^+-rich (**b**, **d**) buffer solutions. The UV-monitored thermal curves were recorded following the signal at 295 (**a**, **b**) or 260 (**c**, **d**) nm in both saline conditions, with a temperature scan rate of 1 °C/min.

Aptamer	UV-melting and annealing experiments					
	K^+-rich buffer			Na^+-rich buffer		
λ = 295 nm	Melting T_m (°C)	ΔT_m (°C)	Annealing T_m (°C)	Melting T_m (°C)	ΔT_m (°C)	Annealing T_m (°C)
15-mer NU	41	-	42	29	-	29
NU172	42	-	41	41	-	40
cycNU172-EG2	44	+ 2	47	39	- 2	43
cycNU172-EG3	42	0	45	40	- 1	42
cycNU172-Ph	44	+ 2	41	40	- 1	43
cycNU172-Pro	42	0	46	41	0	45
λ = 260 nm	Melting T_m (°C)	ΔT_m (°C)	Annealing T_m (°C)	Melting T_m (°C)	ΔT_m (°C)	Annealing T_m (°C)
NU172	60	-	47	58	-	48
cycNU172-EG2	71	+ 11	58	62	+ 4	55
cycNU172-EG3	71	+ 11	60	71	+ 13	50
cycNU172-Ph	68	+ 8	50	64	+ 6	44
cycNU172-Pro	71	+ 11	55	68	+10	54

Table S2. Apparent melting temperature values obtained by UV-monitored thermal experiments at 295 and 260 nm for heating and cooling profiles of the here studied oligonucleotides in the selected K^+- and Na^+-rich buffer solutions. Apparent T_m values were determined from the normalized data as the temperature at which half of the sample is folded, *i.e.* NΔA = 0.5. The error associated with the T_m determination is ± 1 °C. ΔT_m is calculated by subtracting the measured T_m of unmodified NU172 from that observed for each cyclic NU172 analogue.

Aptamer	CD profile					
	K^+-rich buffer			Na^+-rich buffer		
	+	-	+	+	-	+
**15-mer NU**	294	269	248	294	276	257
**NU172**	293	262	245	293	261	247
**cycNU172-EG2**	292	263	245	292	260	247
**cycNU172-EG3**	292	262	246	291	260	247
**cycNU172-Ph**	291	259	245	290	258	246
**cycNU172-Pro**	293	262	245	292	260	246

Table S3. Specific λ values in nm relative to the maxima and minima of the observed CD bands of the analyzed oligonucleotides. "+" and "–" refer to the positive and negative CD bands, respectively.

15-mer NU	Prediction of G4 topologies relative abundance from SVD analysis of CD spectra	
K^+-rich buffer		
Parallel (%)	Hybrid (%)	Antiparallel (%)
0	17	83
Na^+-rich buffer		
Parallel (%)	Hybrid (%)	Antiparallel (%)
36	0	63

Table S4. Prediction of the relative abundance of the G4 topologies adopted by the 15-mer NU, obtained by singular value decomposition (SVD) analysis of the CD spectra recorded in both the selected buffer solutions, performed by exploiting the software developed by del Villar-Guerra *et al.*(2) Deviations from 100 % (± 1 %) are due to significant digits approximation of the values originally obtained by the simulations.

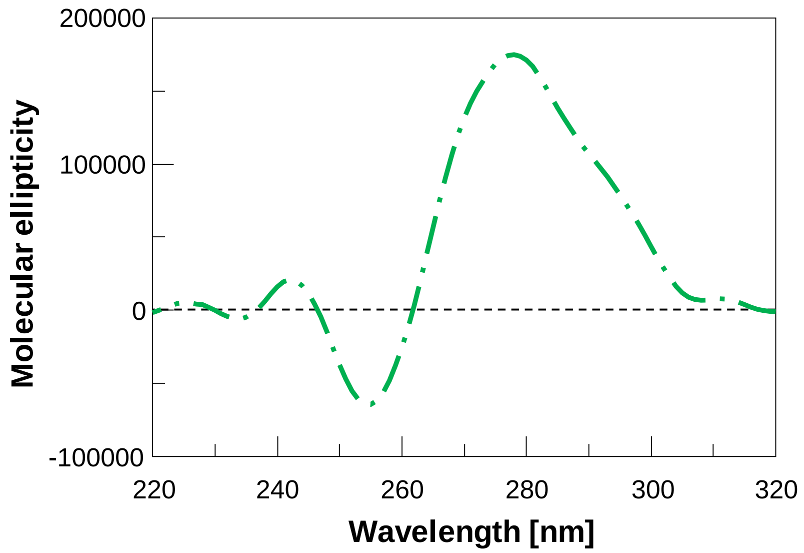

Figure S15. CD difference spectrum obtained subtracting the spectrum of the 15-mer NU from that of NU172 in the selected K^+-rich buffer solution.

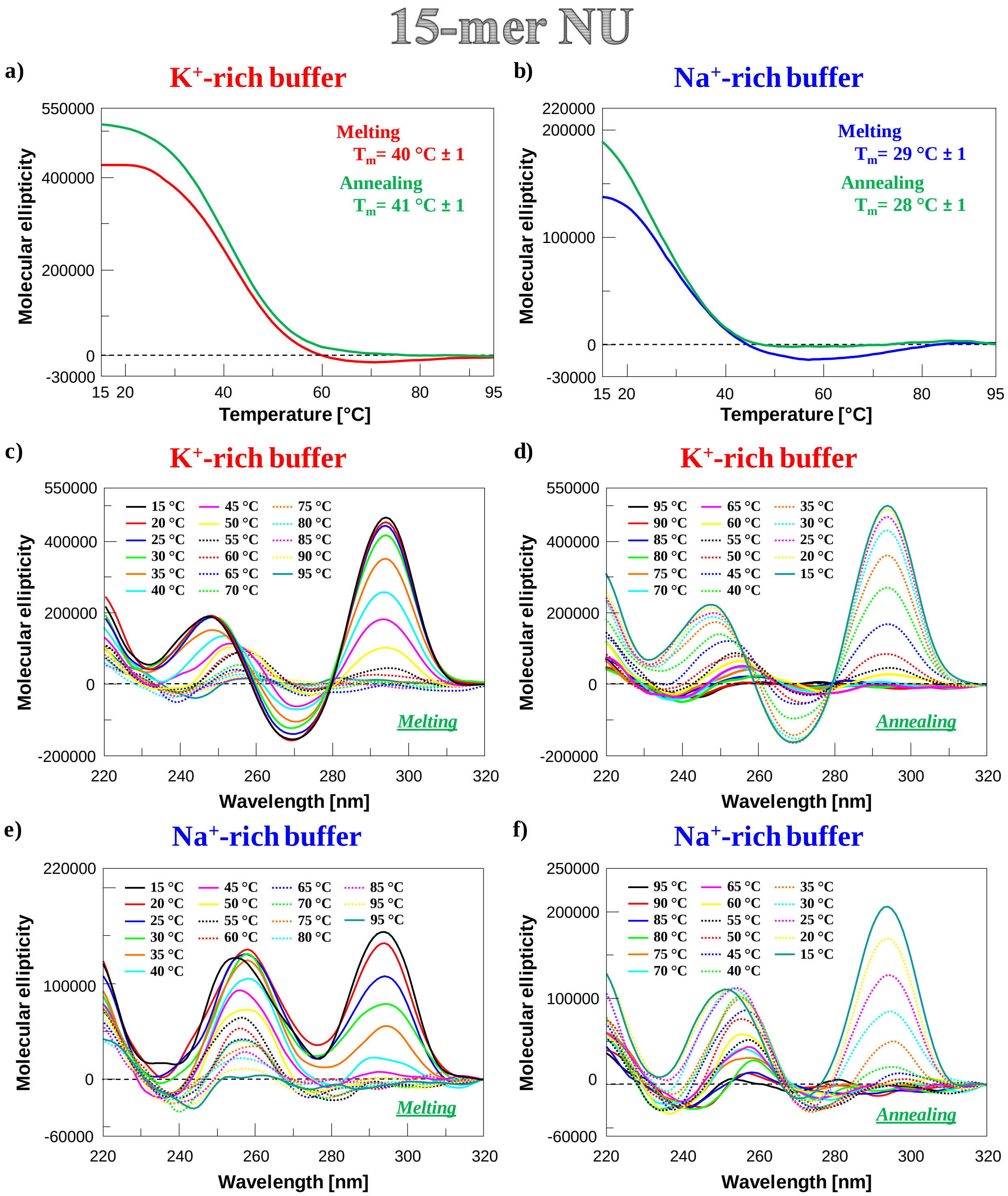

Figure S16. CD-melting and CD-annealing profiles (**a**, **b**) of the 15-mer NU at 2 μM concentration in both the selected K^+- (**a**) and Na^+-rich (**b**) buffer solutions. The CD curves were recorded following the CD signal at 294 nm in both saline conditions, with a temperature scan rate of 1 °C/min. Overlapped CD spectra of 15-mer NU recorded every 5 °C during the melting (**c**, **e**) and cooling processes (**d**, **f**) in both the selected K^+- (**c**, **d**) and Na^+-rich (**e**, **f**) buffer solutions.

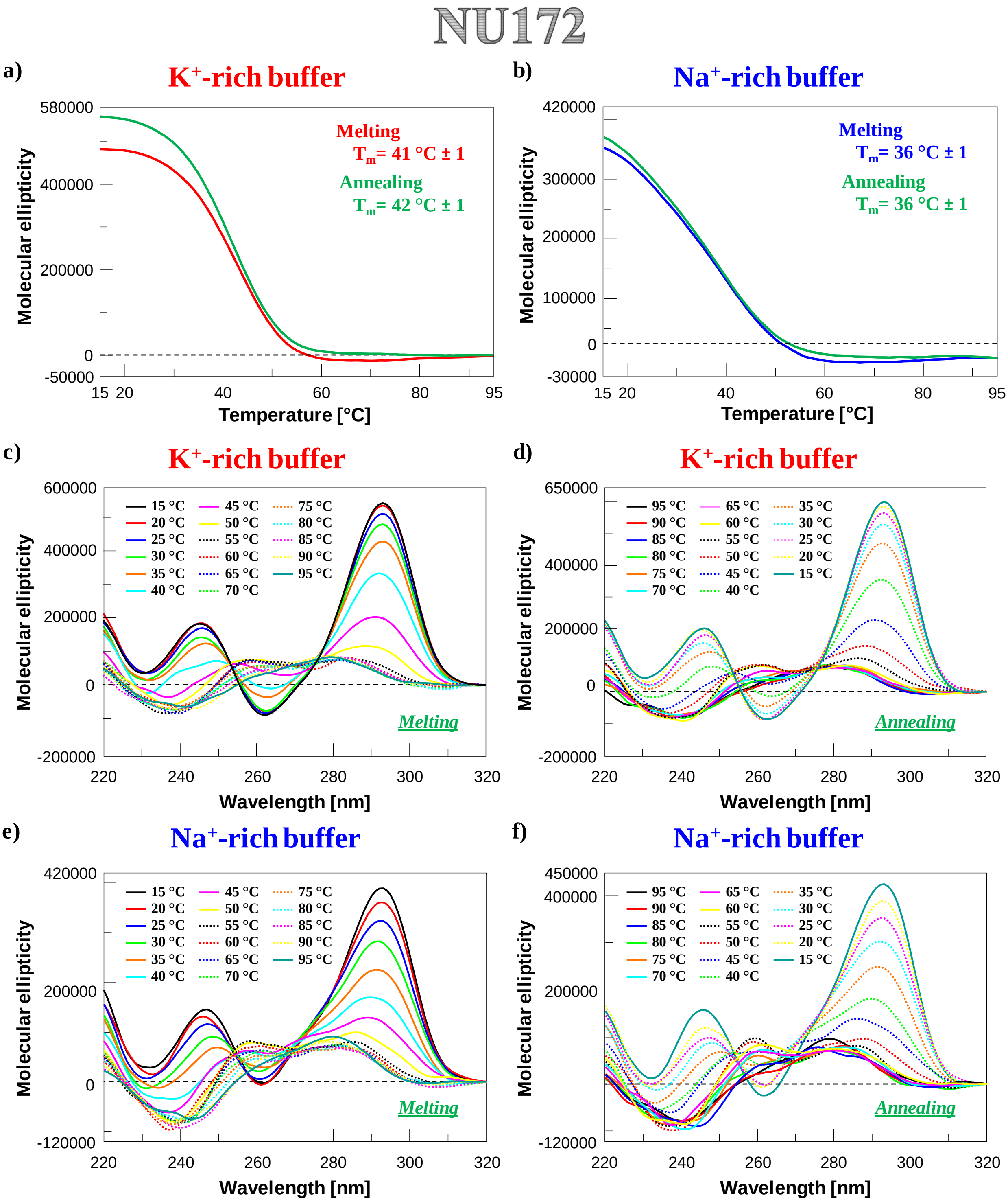

Figure S17. CD-melting and CD-annealing profiles (**a**, **b**) of NU172 at 2 μM concentration in both the selected K^+- (**a**) and Na^+-rich (**b**) buffer solutions. The CD curves were recorded following the CD signal at 293 nm in both saline conditions, with a temperature scan rate of 1 °C/min. Overlapped CD spectra of NU172 recorded every 5 °C during the melting (**c**, **e**) and cooling processes (**d**, **f**) in both the selected K^+- (**c**, **d**) and Na^+-rich (**e**, **f**) buffer solutions.

cycNU172-EG2

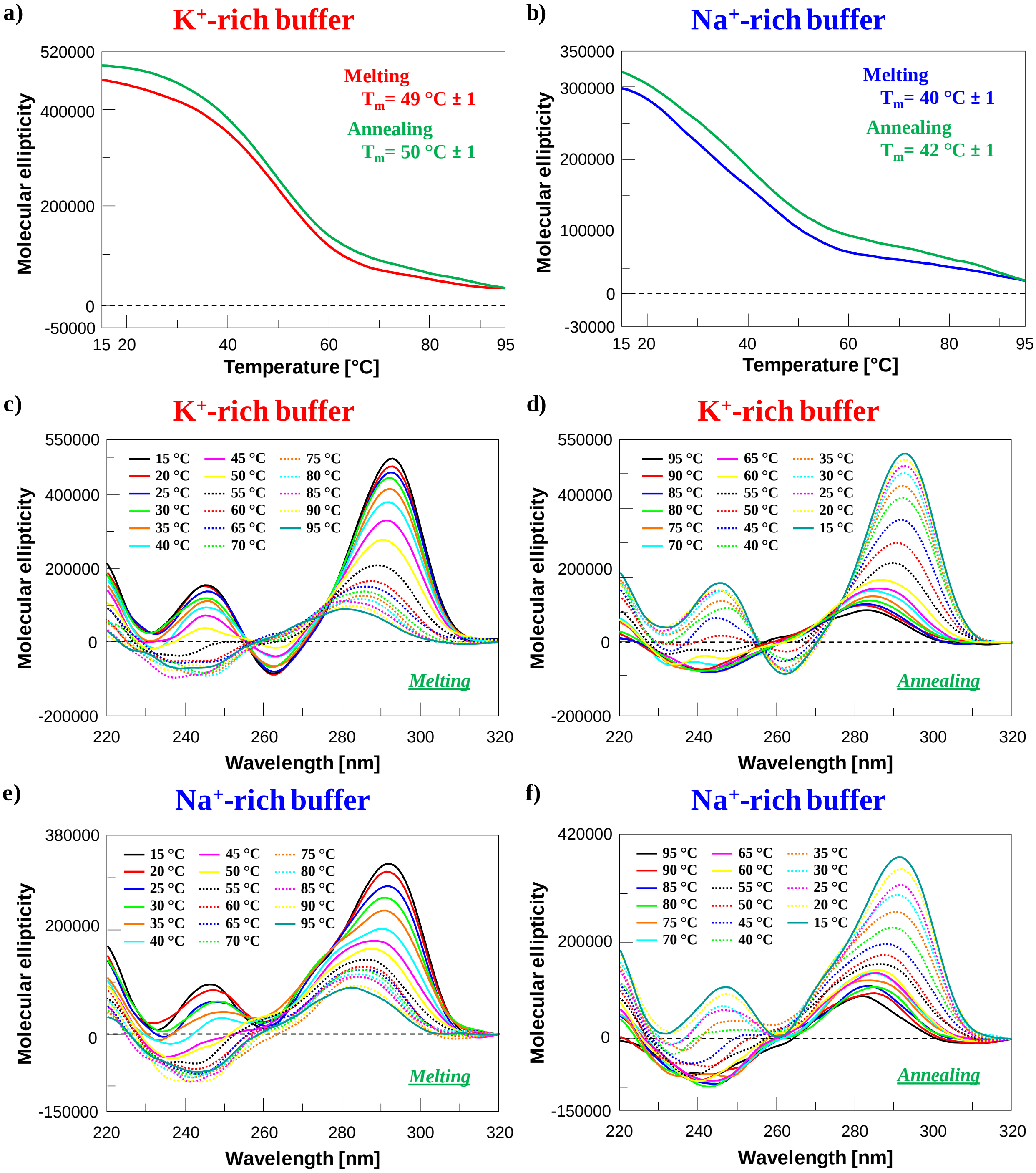

Figure S18. CD-melting and CD-annealing profiles (**a**, **b**) of **cycNU172-EG2** at 2 µM concentration in both the selected K^+- (**a**) and Na^+-rich (**b**) buffer solutions. The CD curves were recorded following the CD signal at 292 nm in both saline conditions, with a temperature scan rate of 1 °C/min. Overlapped CD spectra of **cycNU172-EG2** recorded every 5 °C during the melting (**c**, **e**) and cooling processes (**d**, **f**) in both the selected K^+- (**c**, **d**) and Na^+-rich (**e**, **f**) buffer solutions.

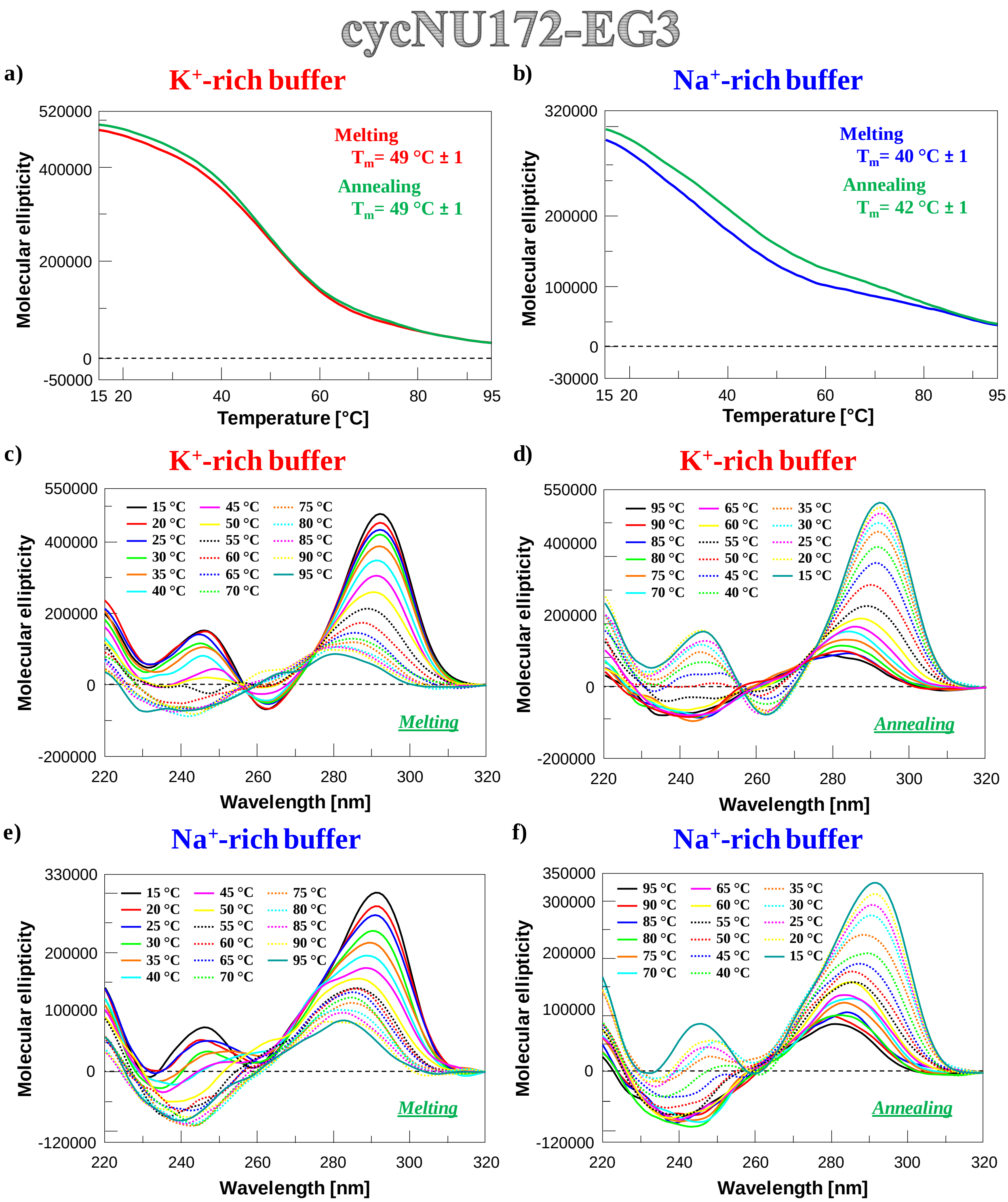

Figure S19. CD-melting and CD-annealing profiles (**a**, **b**) of **cycNU172-EG3** at 2 µM concentration in both the selected K^+- (**a**) and Na^+-rich (**b**) buffer solutions. The CD curves were recorded following the CD signal at 292/291 nm respectively in the K^+/Na^+ buffer solutions, with a temperature scan rate of 1 °C/min. Overlapped CD spectra of **cycNU172-EG3** recorded every 5 °C during the melting (**c**, **e**) and cooling processes (**d**, **f**) in both the selected K^+- (**c**, **d**) and Na^+-rich (**e**, **f**) buffer solutions.

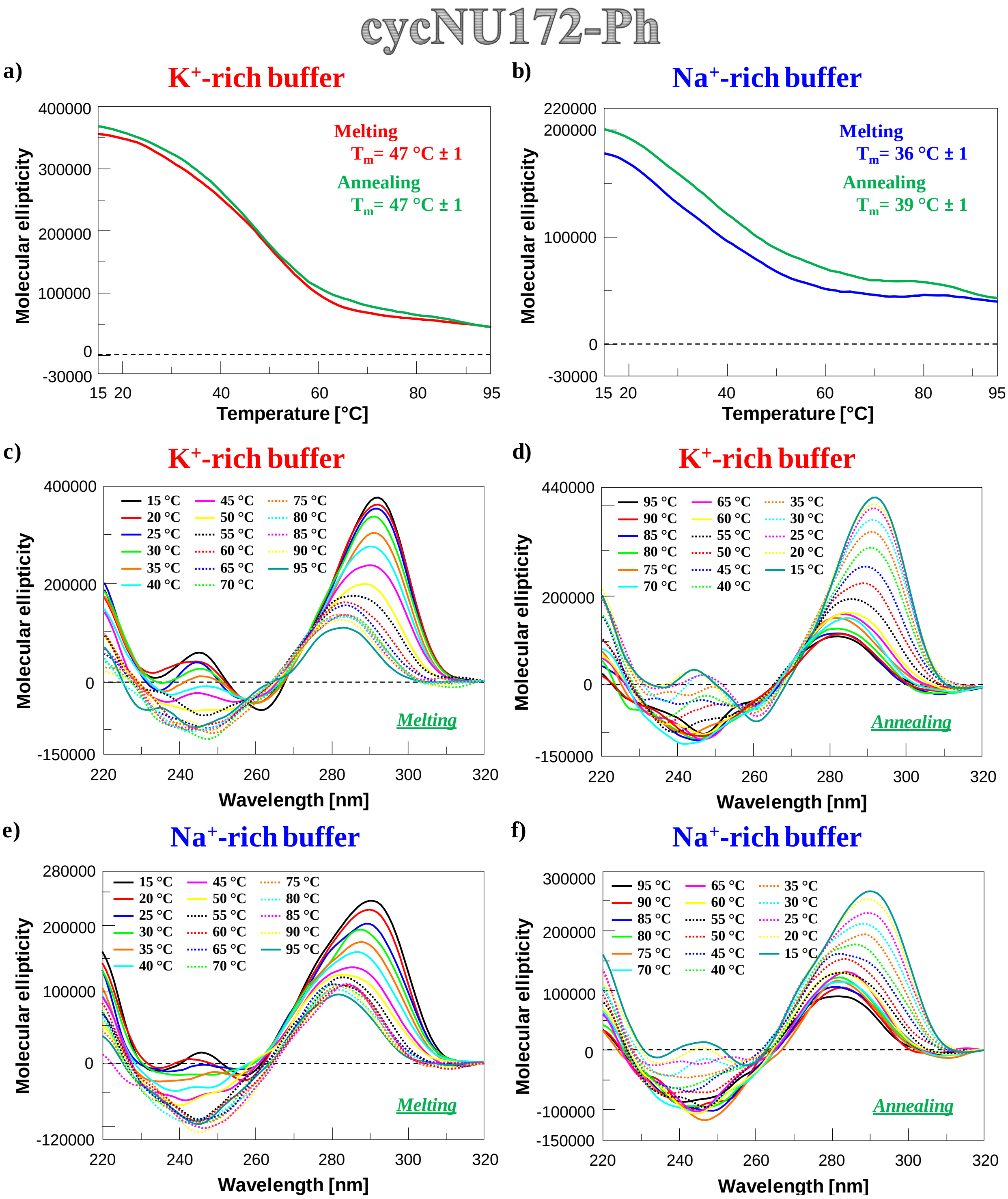

Figure S20. CD-melting and CD-annealing profiles (**a**, **b**) of **cycNU172-Ph** at 2 µM concentration in both the selected K^+- (**a**) and Na^+-rich (**b**) buffer solutions. The CD curves were recorded following the CD signal at 291/290 nm respectively in the K^+/Na^+ buffer solutions, with a temperature scan rate of 1 °C/min. Overlapped CD spectra of **cycNU172-Ph** recorded every 5 °C during the melting (**c**, **e**) and cooling processes (**d**, **f**) in both the selected K^+- (**c**, **d**) and Na^+-rich (**e**, **f**) buffer solutions.

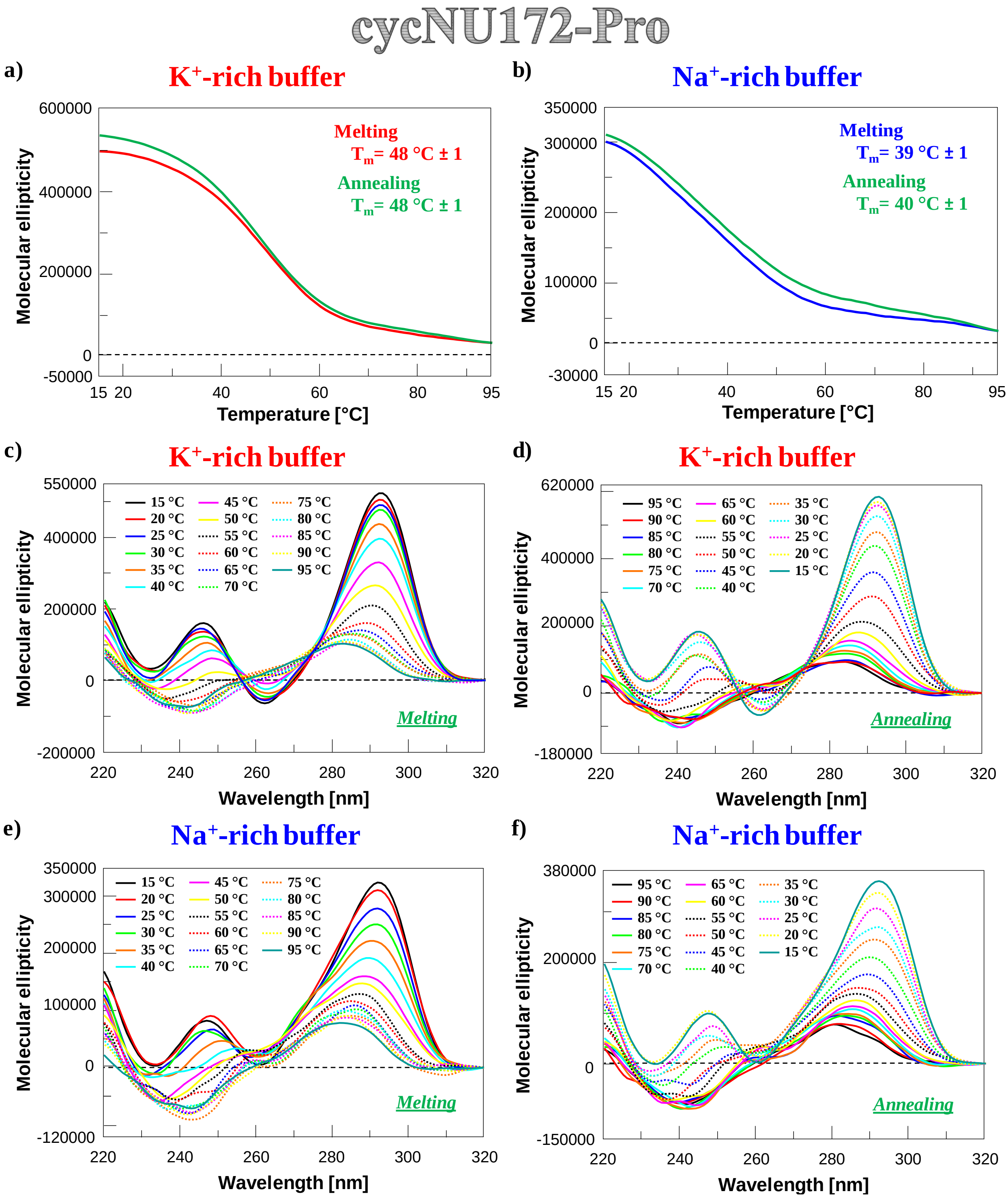

Figure S21. CD-melting and CD-annealing profiles (**a**, **b**) of **cycNU172-Pro** at 2 µM concentration in both the selected K^+- (**a**) and Na^+-rich (**b**) buffer solutions. The CD curves were recorded following the CD signal at 293/292 nm respectively in the K^+/Na^+ buffer solutions, with a temperature scan rate of 1 °C/min. Overlapped CD spectra of **cycNU172-Pro** recorded every 5 °C during the melting (**c**, **e**) and cooling processes (**d**, **f**) in both the selected K^+- (**c**, **d**) and Na^+-rich (**e**, **f**) buffer solutions.

Aptamer	CD-melting and annealing experiments					
	K^+-rich buffer			Na^+-rich buffer		
	Melting T_m (°C)	ΔT_m (°C)	Annealing T_m (°C)	Melting T_m (°C)	ΔT_m (°C)	Annealing T_m (°C)
**15-mer NU**	40	-	41	29	-	28
**NU172**	41	-	42	36	-	36
**cycNU172-EG2**	49	+ 8	50	40	+ 4	42
**cycNU172-EG3**	49	+ 8	49	40	+ 4	42
**cycNU172-Ph**	47	+ 6	47	36	0	39
**cycNU172-Pro**	48	+ 7	48	39	+ 3	40

Table S5. Apparent melting temperature values obtained by CD-monitored thermal experiments for heating and cooling profiles of the here studied oligonucleotides in the selected K^+- and Na^+-rich buffer solutions. Apparent T_m values were determined from the normalized data as the temperature at which half of the sample is folded, *i.e.* $\alpha = 0.5$. Each T_m value is calculated as the average of three independent measurements. The error associated with the T_m determination is ± 1 °C. ΔT_m is calculated by subtracting the measured T_m of unmodified NU172 from that observed for each cyclic NU172 analogue.

	CD		
	ΔH^0 (kJ mol^{-1})	ΔS^0 (kJ mol^{-1} K^{-1})	ΔG^0_{298K} (kJ mol^{-1})
**15-mer NU**	-125 ± 2	-0.40 ± 0.01	-7.0 ± 0.5
**NU172**	-170 ± 7	-0.54 ± 0.02	-10.1 ± 0.7
**cycNU172-EG2**	-136 ± 4	-0.42 ± 0.01	-7.8 ± 0.4
**cycNU172-EG3**	-118 ± 4	-0.36 ± 0.02	-9.3 ± 0.2
**cycNU172-Ph**	-120 ± 6	-0.37 ± 0.02	-9.5 ± 0.7
**cycNU172-Pro**	-131 ± 8	-0.40 ± 0.03	-9.9 ± 0.7

Table S6. Standard thermodynamic parameters as derived from van't Hoff analysis (ΔH^0, ΔS^0 and ΔG^0 calculated at 298 K) for the unfolding process of the 15-mer NU, NU172 as well as its cyclic variants, followed by CD spectroscopy in the selected K^+-rich buffer.

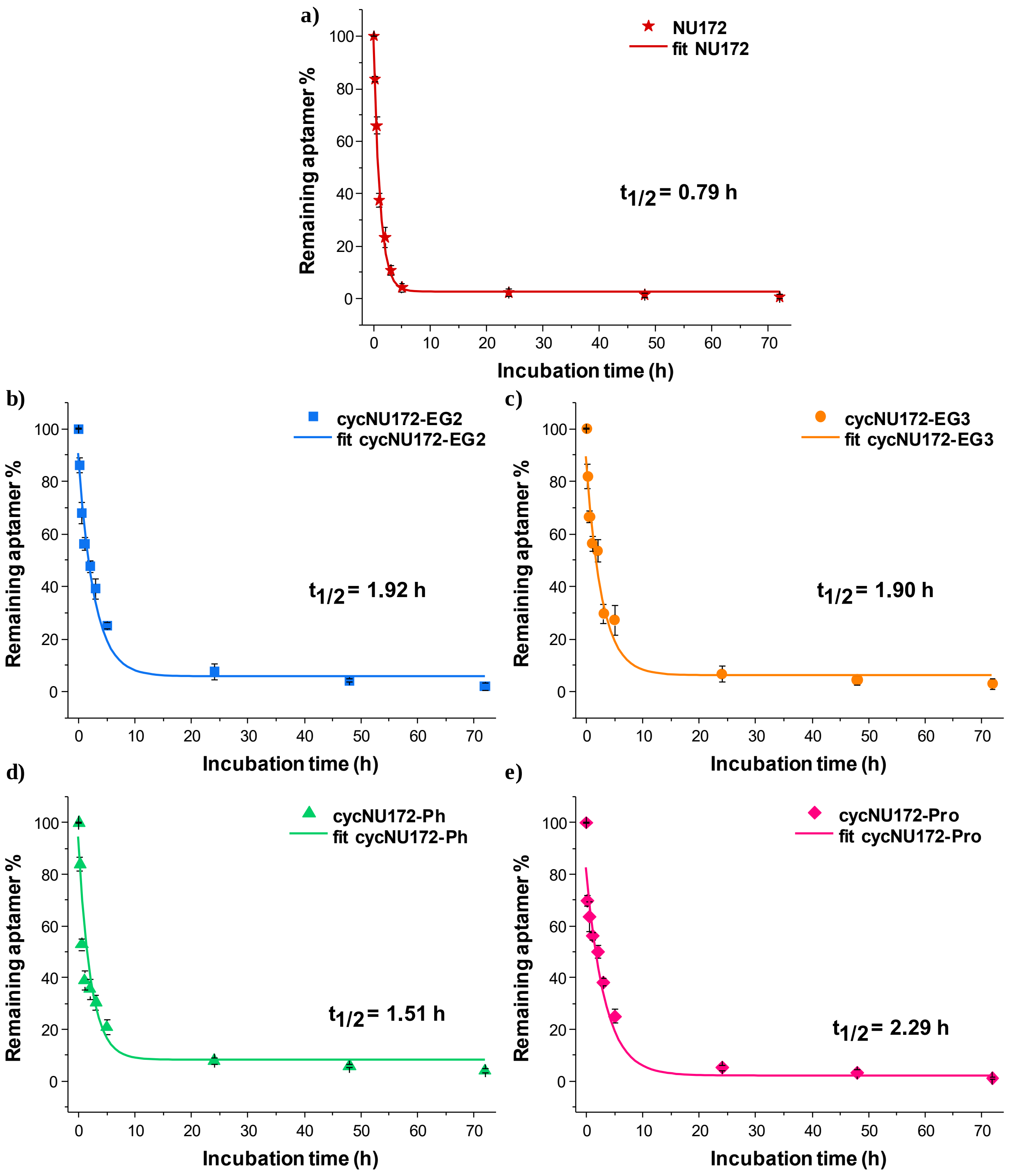

Figure S22. Enzymatic resistance experiments performed on NU172 (**a**) and cycNU172s (**b-e**) incubated in 80 % fetal bovin serum (FBS) as monitored by denaturing 20 % polyacrylamide gel electrophoresis up to 72 h (time points: 0, 0.2, 0.5, 1, 2, 3, 5, 24, 48 and 72 h). Intensity of each oligonucleotide band on the gel is expressed as percentage of the remaining aptamer with respect to the initial one (untreated oligonucleotide) for all the analyzed time points. Data are reported as mean values ± SD (error bars) for multiple independent determinations (at least 5). Obtained values were also fitted with an equation for first order kinetics (lines) allowing to calculate the half-life in serum of each aptamer ($t_{1/2}$).

References:

1. Karsisiotis,A.I., Hessari,N.M.A., Novellino,E., Spada,G.P., Randazzo,A. and Webba da Silva,M. (2011) Topological characterization of nucleic acid G-Quadruplexes by UV absorption and circular dichroism. *Angew. Chem. - Int. Ed. Eng.*, **50**, 10645–10648.

2. Del Villar-Guerra,R., Trent,J.O. and Chaires,J.B. (2018) G-quadruplex secondary structure obtained from circular dichroism spectroscopy. *Angew. Chem. - Int. Ed. Eng.*, **57**, 7171–7175.
